# Distinct Transcriptional Networks in Quiescent Myoblasts: A Role for Wnt Signaling in Reversible vs. Irreversible Arrest

**DOI:** 10.1371/journal.pone.0065097

**Published:** 2013-06-03

**Authors:** Sindhu Subramaniam, Prethish Sreenivas, Sirisha Cheedipudi, Vatrapu Rami Reddy, Lingadahalli Subrahmanya Shashidhara, Ravi Kumar Chilukoti, Madhavi Mylavarapu, Jyotsna Dhawan

**Affiliations:** 1 CSIR-Centre for Cellular and Molecular Biology, Hyderabad, India; 2 Institute for Stem Cell Biology and Regenerative Medicine, Bangalore, India; The Chinese University of Hong Kong, China

## Abstract

Most cells in adult mammals are non-dividing: differentiated cells exit the cell cycle permanently, but stem cells exist in a state of reversible arrest called quiescence. In damaged skeletal muscle, quiescent satellite stem cells re-enter the cell cycle, proliferate and subsequently execute divergent programs to regenerate both post-mitotic myofibers and quiescent stem cells. The molecular basis for these alternative programs of arrest is poorly understood. In this study, we used an established myogenic culture model (C2C12 myoblasts) to generate cells in alternative states of arrest and investigate their global transcriptional profiles. Using cDNA microarrays, we compared G_0_ myoblasts with post-mitotic myotubes. Our findings define the transcriptional program of quiescent myoblasts in culture and establish that distinct gene expression profiles, especially of tumour suppressor genes and inhibitors of differentiation characterize reversible arrest, distinguishing this state from irreversibly arrested myotubes. We also reveal the existence of a tissue-specific quiescence program by comparing G_0_ C2C12 myoblasts to isogenic G_0_ fibroblasts (10T1/2). Intriguingly, in myoblasts but not fibroblasts, quiescence is associated with a signature of Wnt pathway genes. We provide evidence that different levels of signaling via the canonical Wnt pathway characterize distinct cellular states (proliferation vs. quiescence vs. differentiation). Moderate induction of Wnt signaling in quiescence is associated with critical properties such as clonogenic self-renewal. Exogenous Wnt treatment subverts the quiescence program and negatively affects clonogenicity. Finally, we identify two new quiescence-induced regulators of canonical Wnt signaling, Rgs2 and Dkk3, whose induction in G_0_ is required for clonogenic self-renewal. These results support the concept that active signal-mediated regulation of quiescence contributes to stem cell properties, and have implications for pathological states such as cancer and degenerative disease.

## Introduction

Most cells in adult mammals do not divide. During tissue formation, cells exit the cell cycle either permanently or temporarily: for example, skeletal muscle although largely composed of post-mitotic myofibers, harbours rare dormant satellite stem cells. During regeneration of damaged muscle, satellite cells (SC) break quiescence and return to active proliferation. Subsequently, the type of cell cycle exit undertaken by the SC progeny has different consequences. One pathway is co-ordinated with the activation of tissue-specific genes and fusion to form multinucleate contractile myofibers, which restore tissue form and function but cannot return to active cell division. The other pathway, associated with suppression of differentiation, is a transient exit that permits replenishment of the SC reserve and is therefore central to SC function [1]. The two distinct modes of cell cycle exit have implications for maintaining the balance of cell types (differentiated vs. stem cell) in adult tissues, and deregulation of cellular quiescence programs may underlie pathological states such as cancer and degenerative disease.

Adult stem cells cycle rarely (classically revealed in label retention assays), and may spend much of their lifespan in G_0_, yet quiescence is the least understood aspect of the cell cycle. Reversibility of G_0_ requires programs beyond those that control the cell cycle per se [2] [3]. Growing evidence suggests that exit into G_0_ is not a default state resulting from an absence of growth promoting signals, but is actively regulated [4]. The mechanisms that regulate the quiescence program are likely to operate not only at the level of signaling, but also transcriptional and chromatin modulation to maintain cellular identity.

The core transcriptional program of quiescence has been defined in hematopoietic SC [5] [6] and fibroblasts [2]. As with the cell division cycle [7], analysis of G_0_ in yeast [8] [9] provides a conserved framework for understanding quiescence in mammalian cells. Distinct pathways may control the entry into and exit from quiescence [8] [10] and control of this dormant state is emerging as a complex program, with implications beyond arrest [2]. Thus, quiescence-induced programs may include a novel class of tumor suppressor genes that not only enforce cell cycle exit but also control other attributes of ‘hibernating’ cells. Beyond promoting survival under conditions of reduced/altered metabolic activity, quiescence factors would ideally also maintain a state of signal-responsiveness for cell cycle re-entry. Quiescent adult muscle stem cells would need to suppress overt differentiation, yet maintain lineage memory so as to follow the appropriate tissue-specific pathway when activated [11].

Molecular correlates of quiescence are difficult to study comprehensively in vivo: while expression profiling of freshly isolated muscle SC has been reported [12] [13], the G_0_ state itself has not been accessed, as isolation from their niche breaks quiescence, forcing cells to enter G_1_. The current understanding of quiescence biology has benefited from culture systems that generate homogeneous populations of reversibly arrested muscle cells or “G_0_ myoblasts” [1].

Mitogen deprivation of asynchronous C2C12 cultures triggers irreversible cell cycle arrest, fusion and differentiation into multinucleated myotubes, [14] [15]. In serum-deprived cultures, a small proportion (∼20%) of cells resist differentiation and enter reversible quiescence while suppressing MyoD expression [16]. These ‘reserve cells’ have been used to model satellite cell growth control, but little is known about their formation under conditions that cause differentiation of the bulk of myoblasts. By contrast, during suspension culture, >99% of myoblasts exit the cell cycle in an undifferentiated state despite the presence of saturating concentrations of growth factors [17] [18]. Importantly, this anchorage-dependent arrest is synchronously reversed upon restoration of surface contacts in >98% of cells. Our previous studies have demonstrated that reversibly arrested C2C12 myoblasts model several aspects of satellite cell behavior [17] [18]. Firstly, myoblasts arrest in G_0_ as evidenced by the absence of DNA synthesis, a 2C DNA content and suppression of growth-associated genes. Secondly, expression of MyoD and Myf5 is suppressed and differentiation-dependent genes such as Myogenin, myosin, and muscle creatine kinase are not induced. Thirdly, arrested myoblasts are synchronously activated out of G_0_, sequentially express MyoD and Myf5 in G_1_ and enter S phase. Finally, in addition to the myogenic regulators, several genes implicated in SC arrest [19], commitment [20], and activation [21] [1] are similarly regulated during reversible arrest in culture. Taken together, these findings indicate that reversible arrest in suspension culture involves the regulation of some key genes implicated in satellite cell function in vivo [18] [1] Further, we have used this homogenously arrested, synchronized system to identify genes that are induced in G_0_ [18] [22].

Two important concepts emerged from these studies. Firstly, genes such as the RNA-binding protein TTP and the chemokine LIX which were strongly up-regulated in quiescent C2C12 myoblasts are expressed in Pax7+ satellite cells in normal skeletal muscle in vivo [18]. Secondly, genes identified in C2C12 myoblasts on the basis of quiescence-induced expression also function in key regulatory pathways: for example, knockdown of p8/Nupr1 a small chromatin architectural factor induced in G_0_ myoblasts leads to hastening of the cell cycle, supporting an anti-proliferative role [22]; while knockdown of MLL5, a histone methyltransferase induced in G_0_ myoblasts leads to loss of myogenic memory and failure of differentiation upon return to the cell cycle [11]. Thus, substantial evidence supports the hypothesis that genes induced in suspension-arrested G_0_ C2C12 myoblasts are functionally involved in the quiescence program, and warrants further exploration at a global level.

Transcriptomal profiling of quiescent fibroblasts, lymphocytes and HSC [2], [4], [5] have substantially increased our understanding of the global networks that control this important “out-of-cycle” state. Despite the identification of common core programs in G_0_, expectedly featuring tumor suppressors and inhibitors of differentiation, direct comparisons of different cell types in quiescence have not been reported, leaving a gap in our understanding of key mechanisms. Further, no direct comparisons have been made of a single cell type induced to different states of arrest. To address these open questions, we have used closely related cell types (myoblast and fibroblast), as well as compared a single cell type in different states of arrest (reversible vs. irreversible).

In this study, we describe gene expression profiling of reversibly arrested C2C12 myoblasts and C3H 10T1/2 fibroblasts in culture. We define a signature of genes associated specifically with G_0_ and by comparison of G_0_ in two different cell types, reveal the existence of a tissue-specific quiescence program. We also distinguish reversible quiescence in myoblasts from post-mitotic arrest in myotubes. Our analysis shows that Wnt pathway induction is associated with the quiescent state in myogenic cells, implicates Wnt signaling in the choice between reversible and irreversible arrest/differentiation and finally, identifies two new quiescence-induced regulators of Wnt signaling.

## Materials and Methods

### Cell Culture

C2C12 myoblasts [23] [15], were obtained from H. Blau (Stanford) and sub-clone A2 [18] used in all experiments. Myoblasts were maintained in growth medium (GM: DMEM with 20% FBS). C3H10T1/2 cells (a multipotent mesenchymal line) obtained from H. Blau were maintained in DMEM+10% FBS.

***Differentiation*** was induced in cultures at ∼80% confluence after washing with PBS and incubation in differentiation medium (DM: DMEM with 2% horse serum), replaced daily for 3–5 days.

***G_0_ synchronization*** of myoblasts was achieved using suspension culture as described [18]. Briefly, sub-confluent cultures were harvested and cultured as a single cell suspension (10^5^ cells/ml) in semi-solid DMEM containing 1.3% methyl cellulose, 20% FBS (>98% of cells arrest in G_0_ by 48 hrs). Fibroblasts were suspension-arrested in DMEM+10% FBS containing 1.3% methyl cellulose as described for myoblasts.

***Reserve cells*** were generated by the method described previously [24]. Briefly, dense cultures of myoblasts were incubated in DM for five days. Myotubes were quantitatively removed by mild trypsinization, and remaining (adherent) quiescent mononuclear reserve cells isolated by complete trypsinization and replated for 0–24 hrs.

### Transfection

C2C12 myoblasts plated on cover slips (for imaging) or 60 mm dishes (for FACS) were transfected as previously described [11]. Where indicated, cells were treated with rmWnt3A (50 ng/ml, R&D Systems).

***Luciferase assays*** were performed as described [25] on cells plated in 24 well plates and transfected with Topflash or pGL3luc control plasmids, along with pSV2-lacZ to normalize for transfection efficiency using β-gal assays.

### Immunofluorescence

Cells plated on cover slips were fixed, and processed as described [11] for confocal microscopy (antibody details in Materials and Methods S1). Images were adjusted minimally for brightness and contrast using global settings applied to the whole image and assembled using Adobe Photoshop.

### Microarray Analysis

#### Hybridization

RNA was isolated as described [18] from C2C12 myoblasts proliferating asynchronously (MB), G_0_ synchronized (G_0_ MB), 3 day differentiated myotubes (MT) or G_0_ FB (G_0_FB; C3H10T1/2), Cy3- or Cy5-labelled cDNA synthesised using Superscript II, and used in competitive hybridization experiments as described [11]. Each chip [NIA15K mouse spotted cDNA arrays (University Health Network, Ontario)] contains ∼15,000 mouse cDNAs and ESTs [26], spotted in duplicate along with controls. At least 2 arrays (each with duplicate spots) were used for each sample pair, including a biological replicate and a dye reversal experiment. Thus for each gene ID, data was collected from 4–6 replicate hybridized elements.

#### Array informatics and statistics

A suite of statistical and image analysis programs was used for analysis and the MIAME-compliant data is available at GEO (www.ncbi.nlm.nih.gov/geo/- Accession # GSE33676, GPL14883, GPL14884). Array Vision software was used for feature extraction, background subtraction, detection limit and intensity calculations. Normalization to remove systematic bias was done using locally weighted linear regression (LOWESS), flip-dye analysis was performed using MIDAS software (from TIGR), statistical significance analysis (false discovery rate <4%) was performed using SAM (Stanford Microarray Resource). A cut-off of 1.6-fold (Normalized Log Ratio [NLR] 0.67) was used to designate genes as up- or down-regulated. Hierarchical clustering was done using the TIGR TmeV program; GO annotations and other details for all genes were obtained from the Stanford SOURCE database.

**Northern blot analysis** was performed as described [18] using 10 µg of total RNA and ^32^P-labeled probes generated by PCR of mouse cDNA clones derived from the NIA 15K clone set using SP6 and T7 primers.

#### Cell cycle analysis

Flow cytometric analysis of DNA content was done using propidium iodide staining as described [11] and analysed on a FACS Vantage using CelQuest software.

**Quantitative Real-Time RT-PCR** for transcript analysis was performed on an ABI 7700HT PCR machine as described [11]. A list of primers is provided in Materials and Methods S1.

#### Wnt superarray

1 µg of total RNA isolated from MB, G_0_ MB or MT was analysed by Q-RT-PCR in a pre-spotted 384 well format according to manufacturer’s instructions (Superarray, Qiagen). The superarray contains primers specific to 84 different genes in the Wnt pathway.

#### Knockdown analysis

Myoblasts were transfected with si or shRNAs (control si/sh RNA, Dkk3sh and Rgs2sh) as described [11]. Transfected cells were purified by FACS-sorting (see below) and extent of knockdown confirmed by Q-RT-PCR and western blot analysis. Details of knockdown oligos and primers are available as Materials and Methods S1.

#### Chromatin Immuno-Precipitation (ChIP) assay

ChIP analysis was performed essentially as described earlier [11]. Briefly, C2C12 cells were grown to 80% confluency in GM, washed with PBS and collected in cell dissociation buffer (Sigma), re-suspended in GM and chromatin complexes cross-linked with HCHO (added to a final concentration of 1%). Cross-linking was quenched by addition of PBS, cells were washed by centrifugation through ice-cold PBS, the pellet re-suspended in SDS lysis buffer and processed for sonication. Sonication conditions (available on request) were standardized for different cellular states since nuclear condensation/chromatin configuration differs and sonicated samples stored at −80 as aliquots. Pre-clearing, antibody pull-down, elution, reversal of cross-linking and DNA isolation was performed as per manufacturers instructions (ChIP kit, Millipore). For each IP 2–5 µg of primary antibody was used. Control pull-downs used IgG (5 µg) to assess specificity.

Quantitative Real-time PCR analysis on ChIP samples was performed on an ABI 7900HT cycler (Applied Biosystems) as described [11]. The ct value of input was subtracted from IP and Control IgG sample and the resulting IP value was normalized again with the resulting control IgG value for calculation of fold enrichment in each sample by 2^(−ΔΔct)^ method [ie 2^−[(IP-input)−(IgG−input)]^]. The values obtained for each gene represent normalized fold enrichment of the IP protein over input and Control IgG. These values are represented as fold change with respect to the level in myoblast (Mb) samples, so that different genes and different samples can be compared directly. ChIP primers used for Myogenin cover a genomic region including the well-characterized Mef2 binding site and the −130 bp upstream E-box [27]. The Myf5 ChIP primers were designed to cover the −57 kb upstream epaxial enhancer which is a characterized Myf5 cis regulatory region known to be important for Myf5 expression in limb bud [28]. Details of primers are provided in Materials and Methods S1.

#### Colony assays (CFU)

Control and treated MB were held in suspension for 48 hrs (with or without Wnt3a (R&D systems cat# 1324-WN) or sFRP2 (gift from Dr. Arun Dharmarajam, School of Anatomy & Human Biology The University of Western Australia), recovered from methocel, counted, re-suspended in GM without factors, plated at clonal density (400 cells/150 mm dish) and cultured for 7 days. Colonies were stained with methylene blue for counting. shRNA transfected cells were identified using a co-transfected EGFPC1 (Invitrogen, USA) plasmid, GFP+ cells were FACS-sorted and analysed by plating in CFU assays.

## Results and Discussion

### Model System

**Figure 1:**
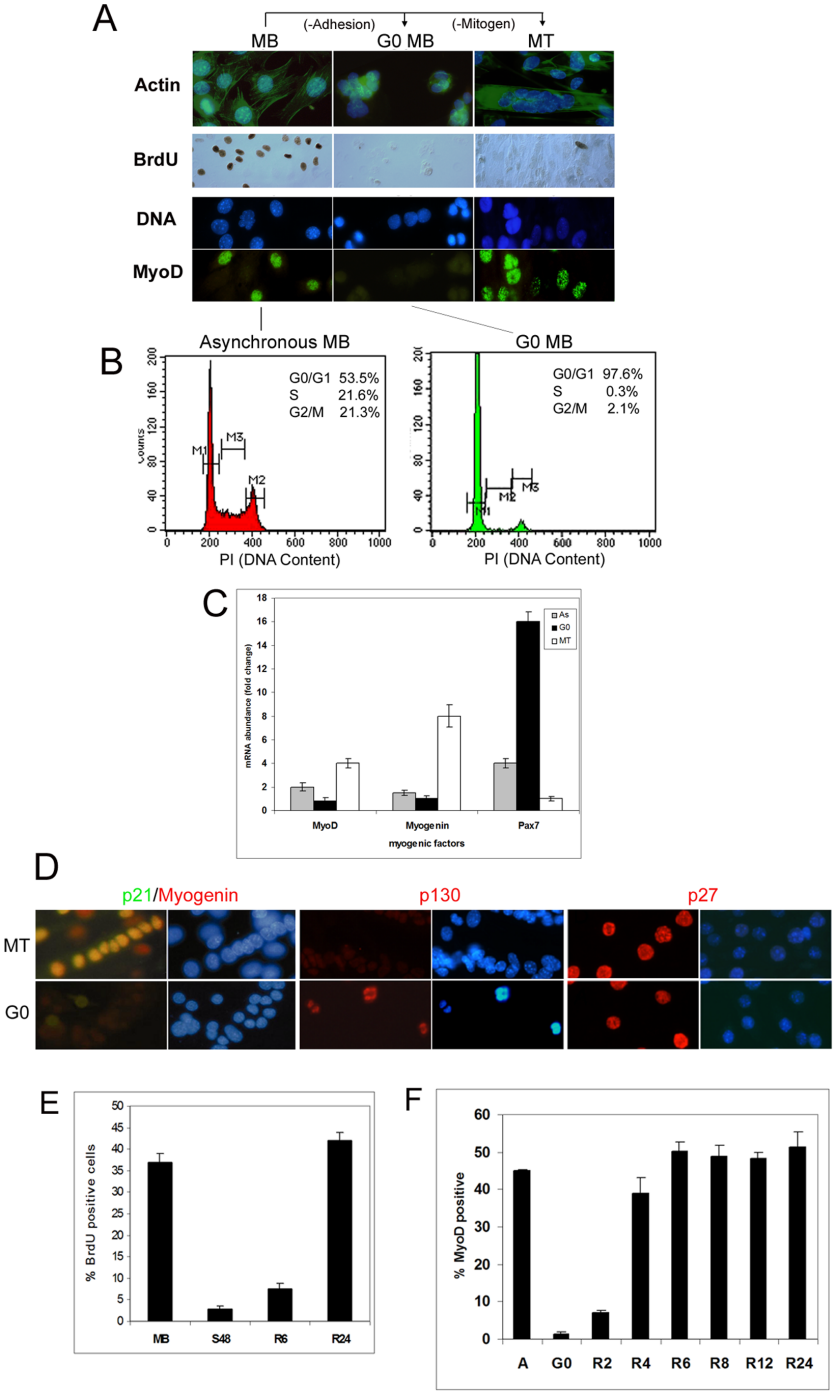
C2C12 myoblasts enter alternate states of arrest. (**A**) Asynchronous proliferating myoblasts (MB) were held in suspension in mitogen-rich media to induce quiescence (arrest in an undifferentiated mono-nucleated state; G_0_ MB), or shifted to mitogen-poor medium for 96 hrs to induce differentiation (arrest coupled with fusion into multinucleated myotubes; MT). Actin staining (Oregon Green-Phalloidin, top panel) reveals the distinct morphology of these 3 states. Nuclei are detected with Hoechst 33352. DNA synthesis was analyzed by detection of BrdU incorporated in a 15-minute pulse (middle panel)- ∼40% of cycling MB are labeled, while less than 1% of G_0_ MB synthesize DNA. In differentiated cultures (MT), only residual mono-nucleated cycling cells (5–10%) incorporate label whereas myotube nuclei do not. Expression of determination factor MyoD can be detected in ∼50% of proliferating MB, is lost in G_0_ but sustained in MT (lower panel). (**B**) FACS analysis of DNA content of cycling (Asynchronous MB) and 48-hr suspension-cultured populations confirms that >97% of cells in suspension (G_0_ MB) possess a 2C DNA content while >40% of cells in an asynchronous culture have replicated their genomes (representative graphs from 4 independent experiments). (**C**) Muscle transcription factor expression distinguishes alternate states of arrest. Q-RT-PCR analysis of specification/survival factor Pax7, determination factor MyoD, and early differentiation marker Myogenin (MyoG) in asynchronous proliferating myoblasts (As), suspension-arrested MB (G_0_) and differentiated MT (MT). Values represent normalized fold differences between GAPDH (control) mRNA and myogenic mRNAs in each sample (n = 3). (**D**) Cell cycle inhibitor expression distinguishes alternate states of arrest-G_0_ MB and MT express different combinations of cyclin-dependent kinase inhibitors p21/p27 and Rb-related p130. Immuno-detection of p21, p130 or p27 in MT and G_0_; p21 (green) co-localizes with MyoG (red) in all nuclei of differentiated MT but neither factor is expressed in G_0_; p130 is specific to G_0_ and p27 is expressed in both G_0_ and MT. Data depicted is representative of three independent experiments. (**E**) G_0_ arrest is reversible. DNA synthesis in asynchronous MB (Mb), 48-hour suspension cultures (G_0_) and G_0_ MB replated for 6 or 24 hours (R6, R24). BrdU detected as in Fig. 1A. Values represent mean+SEM (n = 3). (**F**) MyoD expression is suppressed in G_0_ and restored during early reactivation. Asynchronous cultures (A) were arrested in G_0_ (48 hrs) and reactivated by replating for 2 to 24 hrs (R2–R24). MyoD expression detected as in Fig. 1A. Values represent mean+SEM (n = 3).

The term G_0_ is widely used to refer to the non-replicative state in which either post-mitotic cells or reversibly arrested cells exist. Here, we use G_0_ to refer exclusively to reversible arrest. Mitogen deprivation of adherent myoblasts (MB) triggers arrest, fusion, sustained expression of the myogenic regulatory factor (MRF) MyoD, activation of Myogenin (MyoG) and differentiation into multinucleated myotubes (MT) (Fig. 1A) [29]. By contrast, non-adherent culture of MB in mitogen-rich methyl-cellulose induces cell cycle exit in an undifferentiated state, and loss of MyoD expression [17] [18]. These MyoD-negative MB fail to incorporate BrdU (Fig. 1A), possess a 2C DNA content (Fig. 1B), and express Pax7 and specific cell cycle inhibitors (Fig. 1C,D). Differentiated MT enter a post-mitotic state that cannot be reversed by mitogens [14], whereas suspension-arrest is reversed upon replating on adhesive surfaces [17] (Fig. 1E). Several criteria confirm that suspension-arrested MB enter G_0_: absence of DNA synthesis, a G_1_ DNA content (an un-replicated genome), gross transcriptional and translational suppression, absence of both myogenic and growth-associated gene expression, and the extended kinetics of S-phase re-entry in comparison to exponentially cycling populations [18] [17]. Cell cycle re-entry restores differentiation competence, as MyoD is reactivated in G_1_ (Fig. 1F), [25] [30], but overt differentiation needs additional cues [31] [32].

### Microarray Profiling of Arrested Myoblasts and Fibroblasts: Experimental Design and Data Analysis

Our earlier studies [11] [18] distinguished the state of G_0_ from that of differentiated myotubes with respect to individual genes, laying the foundation for a genome-wide analysis.

**Figure 2:**
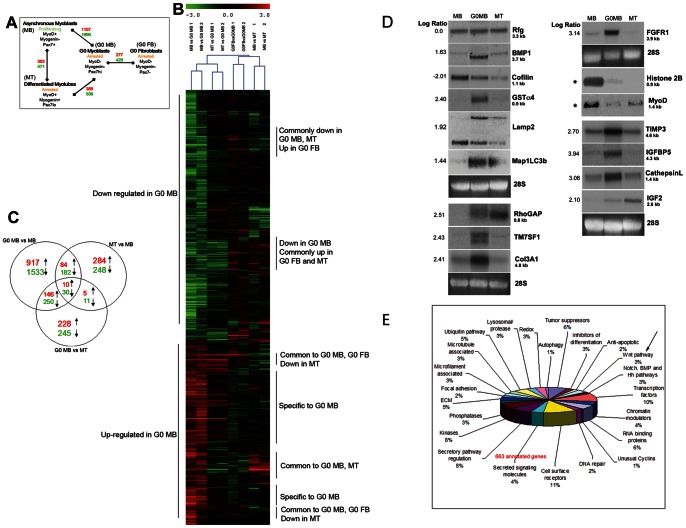
Microarray analysis reveals distinct genetic programs in reversible and irreversible arrest. (**A**) Schematic of microarray loop experiment. RNA isolated from G_0_ MB was analyzed by 2-color competitive hybridization to NIA15K mouse cDNA arrays. Pair-wise analysis: G_0_ MB vs. either proliferating MB, differentiated MT or G_0_ FB. In each pair, genes consistently up- or down-regulated (>1.6 fold, normalized log ratio (NLR) 0.67, false discovery rate 4%) were selected for further analysis. The numbers of genes regulated between each pair of samples are shown: induced (red), suppressed (green). Expression of key regulators characteristic of the different samples is indicated, confirming the identity of each cellular state. (B) Hierarchical clustering (MEV software, TIGR) shows the relatedness of the myoblast and fibroblast sample pairs drawn from independent biological experiments including a dye reversal test. Based on the total transcriptional response, genes up-regulated in G_0_ MB fall into 3 broad categories-genes specifically induced in G_0_ MB (the largest number), genes induced in both G_0_ MB and MT but down-regulated in G_0_ FB, and genes induced in G_0_ MB and G_0_ FB but not in MT. (**C**) In muscle cells, common features of two types of cell cycle exit (reversible vs irreversible) are overshadowed by differences. Venn diagram reveals minimal overlap between the three myogenic sample pairs (MB vs G_0_ MB, MB vs MT, G_0_ MB vs MT), and show that distinct transcriptional programs characterize reversible arrest vs. differentiation-coupled irreversible arrest. (D) Validation of microarray analysis using Northern blots. RNA samples used for microarray analysis were also interrogated with ^32^P-labelled probes representing genes selected to test the range of quantitative changes in expression (i) genes that showed no change (log ratio of 0), (ii) genes that were down-regulated in G_0_ (negative log ratio) or (iii) genes that were up regulated in G_0_ (positive log ratio). Numbers on the left of blots represent log ratios derived from G_0_ vs. asynchronous myoblast array. Gene symbols & transcript sizes are shown on the right of each blot. 28S rRNA was used as a loading control. Rfg, a gene expressed equally in all conditions. Cofilin, a regulator of microfilament dynamics - down-regulated in both G_0_ Mb and in Mt. Of the 12 transcripts selected from the array as enriched in G_0_, all 12 showed G0 induced expression. Of these, 8 (FGFR1, BMP1, GSTa4, TM7SF1, IGFBP5, TIMP3, Cathepsin L, Col3A1) were specifically induced in G_0_ MB, and not in differentiated MT. The other 4 genes, IGF2, Rho GAP, LAMP2 and Map1LC3b were induced in both G_0_ MB and MT. Histone 2B and MyoD transcripts (not derived from array analysis, marked by *) were detected to confirm the cellular state. E. Functional diversity of genes specifically enriched in G_0_ MB indicates a signature of ***recycling*** (autophagy, ubiquitin pathway), ***resistance*** (anti-apoptosis) and ***repair*** (DNA repair). Gene ontology classification of 663 annotated gene from the list of genes upregulated in quiescent (G0) MB suggests that enhanced survival mechanisms may compensate for depressed metabolic functions in quiescence (see Supplementary Information for altered cell cycle and metabolic pathways). In addition, there is evidence of altered expression of genome reprogramming factors (chromatin and transcriptional regulators), signaling (growth factors, GF receptors, signaling adaptors) and developmental regulators [Wnt (indicated by arrow), Hh, BMP, Notch pathways)]. The surprising increase in induction of Wnt genes in quiescence was further investigated.

To directly assess alternate programs of cell cycle exit, we compared reversibly arrested and post-mitotic (differentiated) C2C12 cells by microarray profiling using cDNA arrays (Fig. 2A): To identify transcripts induced in quiescence, we compared G_0_ MB with asynchronously growing MB. To enrich genes induced by cell cycle exit in an undifferentiated state, we compared G_0_ MB with permanently arrested differentiated MT. To enrich genes associated with differentiation, we compared MT with MB. Finally, to explore tissue-specificity in G_0_, we compared G_0_ MB with isogenic G_0_ ‘fibroblasts’ (FB; multipotent C3H10T1/2 cells, which like C2C12, are derived from C3H mice). Microarray data can be accessed at GEO with accession number GSE33676. Genes differentially regulated between the 4 sample pairs are shown in Fig. 2A. Among the 15,000 gene elements surveyed, 1157 were induced >1.6-fold in G_0_ (q value [false discovery rate] 4%), supporting the notion that quiescence involves large-scale reprogramming of the transcriptome.

### Genes Enriched in G_0_ myoblasts Define a Distinct Program of Reversible Arrest that is Overlaid by Tissue-specific Features

Hierarchical clustering (p<0.05) showed that all 3 non-cycling cellular states (G_0_ MB, MT, G_0_ FB) share features, but a distinct program specific to G_0_ MB also emerged (Fig. 2B, C). Compared to growing myoblasts (MB), several classes of genes were discerned in the three arrested samples: (i) specifically up-regulated in G_0_ MB (ii) commonly induced in G_0_ MB and MT (iii) induced to a greater extent in MT than in G_0_ MB (iv) commonly up-regulated in G_0_ MB and G_0_ FB (v) induced in G_0_ FB to a greater extent than in G_0_ MB.

The classic experiments of Davis et al [33] showed that forced expression of MyoD on its own could reprogram C3H10T1/2 fibroblasts to a myogenic fate. Our observations show that despite the loss of MyoD expression in myoblasts that enter G_0_, the quiescence program of these closely related cell types is distinct, indicating a tissue specific layer superimposed on the common program. The overlap between the three arrested states is shown in Fig. 2C.

To independently validate array data, expression of 12 G_0_-induced transcripts was confirmed by northern blot analysis (Fig. 2D). Genes selected represent a range of normalized log ratio values: NLR 0 (unchanged/control), NLR –2.01 (down-regulated in G_0_) and NLR +0.99 to +3.94 (mildly to strongly up-regulated in G_0_). As expected, the magnitude of differential expression varied between the two techniques (arrays vs. blot analysis), but the expression of all 12 genes tested was in concordance, validating the array analysis.

### G_0_-enriched Genes Define a Quiescence Program Beyond Cell Cycle Arrest

Of 1157 transcripts showing a consistent >1.6-fold induction in G_0_ MB, 663 annotated genes were assigned to functional classes using gene ontology (GO) terms and pathway analysis (Fig. 2E). Suppressed proliferation (Fig. S1), and a shift to glycolysis (Table S1F), two defining features of quiescent cells were evident.

G_0_-induced genes may in theory participate in programs beyond those that induce/maintain arrest per se, such as uncoupling differentiation from arrest, preserving lineage memory, activation of G_0_-specific metabolic pathways, promoting survival/repair during arrest or sustaining the capacity for reactivation. Indeed, the set of genes identified as G_0_-enriched were found to represent all these categories (Table S1). Here we focus on three classes of genes-tumor suppressor genes (TSGs), inhibitors of differentiation and the Wnt signaling pathway (Table 1). Induction of TSGs and inhibitors of differentiation confirm the suppression of both proliferative and tissue-specific programs in the quiescent state. However, induction of Wnt signaling in quiescent cells is surprising, as this pathway has been largely implicated in the control of proliferation [34], with additional reports suggesting a role in differentiation [35].

**Table 1:** Selected classes of genes up-regulated in quiescence.

Unigene ID	Name	Symbol	Fold Change
**A. Tumor suppressors**			
Mm.22701	Growth arrest specific 1	Gas1	4.72
Mm.235580	Retinoblastoma-like 2	Rbl2	2.3
Mm.391933	LIM domain containing preferred translocation partner in lipoma	Lpp	1.82
Mm.272722	Tumor suppressor candidate 3	Tusc3	1.82
Mm.333233	RAB7, member RAS oncogene family	Rab7	4.15
Mm.391419	PRKC, apoptosis, WT1, regulator	Pawr	2.19
Mm.240830	Disabled homolog 2	Dab2	5.21
Mm.149438	Mitochondrial tumor suppressor 1	Mtus1	2.7
Mm.100068	Angiomotin	Amot	4.06
Mm.28853	Pituitary tumor-transforming 1 interacting protein	Pttg1ip	3.79
Mm.139926	RNA binding motif protein 6	Rbm6	3.78
Mm.139418	Sestrin 1	Sesn1	4.07
Mm.390461	High mobility group box transcription factor 1	Hbp1	3.83
Mm.24761	Breast cancer metastasis-suppressor 1-like	Brms1l	2.03
Mm.27913	Polybromo 1	Pb1	3.04
Mm.30837	N-myc downstream regulated gene 1	Ndrg1	18.02
Mm.26722	N-myc downstream regulated gene 2	Ndrg2	1.98
Mm.279256	N-myc downstream regulated gene 3	Ndrg3	2.28
Mm.24094	Disrupted in renal carcinoma 2	Dirc2	1.69
Mm.272183	B-cell translocation gene 1, anti-proliferative	Btg1	3.08
Mm.3258	Proliferin related protein	Plfr	2.7
Mm.4261	CD82 antigen	Cd82	1.67
Mm.46233	RAS-like, estrogen-regulated, growth-inhibitor	Rerg	1.66
Mm.356578	Myeloid ecotropic viral integration site 1	Meis1	2.47
Mm.247566	Myeloid ecotropic viral integration site-related gene 1	Mrg1	2.82
Mm.4325	Kruppel-like factor 4	Klf4	3.54
Mm.291595	Kruppel-like factor 9	Klf9	4.43
Mm.29891	Forkhead box O1	Foxo1	2.98
Mm.132238	CREB binding protein	Crebbp	1.63
Mm.1605	Programmed cell death 4	Pdcd4	4.67
Mm.290834	G protein-coupled receptor 56	Gpr56	2.75
Mm.100399	MAD homolog 4	Smad4	2.1
Mm.275044	Lin-9 homolog	Lin9	2.01
Mm.42944	S-phase kinase-associated protein 1A	Skp1a	1.63
Mm.5264	Fasciculation and elongation protein zeta 1 (zygin I)	Fez1	3.38
Mm.157190	High mobility group AT-hook 2	Hmga2	2.55
Mm.332020	PR-domain containing-2	Prdm2	1.6
Mm.27961	Leprecan 1	Lepre1	2.36
**B. Inhibitors of differentiation**			
Mm.276133	LUC7-like 2	Luc7l2	2.05
Mm.425101	EP300 interacting inhibitor of differentiation 1	Eid1	2.51
Mm.1025	Nuclear factor, erythroid derived 2, like 2	Nfe2l2	1.89
Mm.249934	Signal transducer and activator of transcription 3	Stat3	2.34
Mm.306663	Protein inhibitor of activated STAT 1	Pias1	4.25
Mm.275071	Jun proto-oncogene	Jun	2.04
Mm.4364	Interleukin 6 signal transducer	Il6st	2.4
Mm.56769	Decorin	Dcn	7.46
Mm.390461	High mobility group box transcription factor 1	Hbp1	3.83
Mm.238266	Muscleblind-like 2	Mbnl2	2.21
Mm.356578	Myeloid ecotropic viral integration site 1	Meis1	2.47
Mm.247566	Myeloid ecotropic viral integration site-related gene 1	Mrg1	2.82
Mm.209292	Recombining binding protein suppressor of hairless	Rbpsuh	2.69
Mm.20521	Histone deacetylase 3	Hdac3	2.93
Mm.398543	CUG triplet repeat, RNA binding protein 2	Cugbp2	4.22
**C. Wnt Pathway**			
Mm.278444	Transducin-like enhancer of split 1, homolog of Drosophila E(spl)	Tle1	2.51
Mm.7883	Adenomatosis polyposis coli	Apc	2.41
Mm.240830	Disabled homolog 2	Dab2	5.21
Mm.28262	Regulator of G-protein signaling 2	Rgs2	46.05
Mm.22680	Cadherin EGF LAG seven-pass G-type receptor 1	Celsr1	2.58
Mm.255219	Lymphoid enhancer binding factor 1	Lef1	2.25
Mm.55143	Dickkopf homolog 3 (Xenopus laevis)	Dkk3	5.54
Mm.390461	High mobility group box transcription factor 1	Hbp1	3.83
Mm.271854	Low density lipoprotein receptor-related protein 1	Lrp1	2.65
Mm.297906	Frizzled homolog 7	Fzd7	2.11
Mm.214766	Ankyrin repeat domain 6	Ankrd6	1.77
Mm.1367	Wingless-related MMTV integration site 3A	Wnt3a	2.18
Mm.291928	Catenin (cadherin associated protein), beta 1	Ctnnb1	2
Mm.4871	Tissue inhibitor of metalloproteinase 3	Timp3	1.75
Mm.22701	Growth arrest specific 1	Gas1	4.72
Mm.193099	Fibronectin 1	Fn1	13.14
Mm.288474	Secreted phosphoprotein 1	Spp1	5.16
Mm.3433	Extracellular matrix protein 1	Ecm1	1.98
Mm.71682	Calcium binding and coiled coil domain 1	Calcoco1	3.68

**Selected classes of genes up-regulated in quiescence:** Induction of (A) several known tumor suppressors (negative regulators of the cell cycle) and (B) inhibitors of differentiation is expected since quiescence is defined by absence of both proliferation and differentiation. However, induction of a large number of genes involved in the Wnt pathway (C) is surprising, and suggests quiescence-dependent signalling mechanisms.

### Tumor Suppressors are Enriched in G_0_

A diverse set of cell cycle inhibitors, many of which are mutated in tumors showed elevated expression in G_0_. Established tumor suppressor genes induced in G_0_ MB (Table 1A) include chromatin regulators (polybromo1, Klf4, PRDM2, see also Table S1G), inhibitors of transcription (p130, FOXO1, NURD complex, Meis homeodomain proteins), inhibitors of translation (Pcd4), and signaling molecules (Rab7, Dab2). These TSGs were not induced in MT, suggesting that different players inhibit the cell cycle and suppress tumorigenic potential in these two states of arrest. Interestingly, except for FoxO1, which was also induced in G_0_ FB, most TSGs were specifically induced in myoblast quiescence, reinforcing the concept of a cell-type dependent quiescence program.

Suppression of proliferation is attained not only by repressing positive regulators (Cyclins, CDKs), and associated macromolecular metabolism (DNA replication, proliferation related proteins (PCNA, MCMs), RNA & protein synthesis) but also by inducing repressors such as p130, Forkhead proteins & Kruppel-like factors, known to induce quiescence in other lineages [4]. The induction of multiple TSGs suggests a strong program for failsafe inhibition of neoplastic pathways in these temporarily arrested cells.

### Inhibition of Differentiation

#### a) Transcriptional repressors of myogenesis

A central feature of G_0_ MB is the absence of differentiation, while irreversible cell cycle exit, by contrast, is coupled to differentiation. The quiescence program includes genes that suppress entry into alternate “out of cycle” states such as differentiation [3]. Given the suppression of MyoD and lack of MyoG induction, the absence of downstream muscle genes including sarcomeric components is to be expected. However, upstream inhibitors of MyoD were also induced in G_0_ indicating active suppression of determination/differentiation (Table 1B). These include repressors such as luc7-like2, Eid1, SMAD4, RBP-jk, c-jun, Stat3, Pias1, Pias 2, and HBP1, none of which were induced in MT, suggesting fail-safe pathways to protect against precocious differentiation in G_0_. Interestingly, all these inhibitors of differentiation were expressed at similar levels in G_0_ MB and G_0_ FB except for decorin, a TGF-β pathway member. Indeed, Stat3, Pias1 and CUG-2 were enriched in G_0_ FB compared to G_0_ MB, reflecting the resistance of G_0_ FB to MyoD-induced differentiation [2]. Induction of oncogenes such as c-jun (also seen in freshly isolated SCs [12]) lends support to the notion that the quiescence program encompasses functions beyond arrest of the cell cycle.

#### b) Anti-myogenic signaling network

Induction of the anti-proliferative, anti-myogenic TGFβ pathway may aid in establishing and/or maintaining a non-dividing undifferentiated state (Fig. S2). In muscle, TGFβ acts as a potent growth inhibitor and tumor suppressor [36] [37]. We observed that many TGFβfamily receptors (TGFβ receptor 2, BMP receptor 1A, activin receptor 1), co-inhibitory molecules (decorin, Gp130/IL6st) and transcriptional effectors (SMAD4) were specifically induced in G_0_ MB, suggesting that quiescence is initiated or maintained by extrinsic cues. SnoN, a negative regulator of TGFβsignaling [38] was down-regulated in G_0_ MB. TGFβ signaling network components (TGFβr2, SMAD4, TGFβ-induced transcripts TSC-4, TSC-5) were all down-regulated in MT compared to G_0_ MB.

These results highlight distinct gene networks associated with reversible and irreversible arrest.

### Genes Enriched in Quiescent Muscle Stem Cells in vivo are Induced during G_0_ in Culture

C2C12 MB, originally derived from adult muscle satellite cells (SC) [23], have been widely used as a model for SC biology. 72 genes enriched in freshly isolated quiescent SCs [12] were also induced in G_0_ C2C12 cells (Table S2). These include proven tumor suppressors (Gas1, FoxO1, sestrin), unusual cyclins, growth factor receptors (Pdgfr, FGFr), ECM molecules (TIMP3, Cola6), transporters (facilitated glucose transporter) and a number of signaling molecules, including those that induce quiescence, such as TGFβ pathway and stem cell factor LRIG1. Thus, the quiescence program induced in culture recapitulates some important features of quiescent muscle SC, and the commonly induced genes may represent a core, context-independent G0 program.

### Developmental Signaling Pathways and Quiescence –a Wnt Signaling Signature

**Figure 3:**
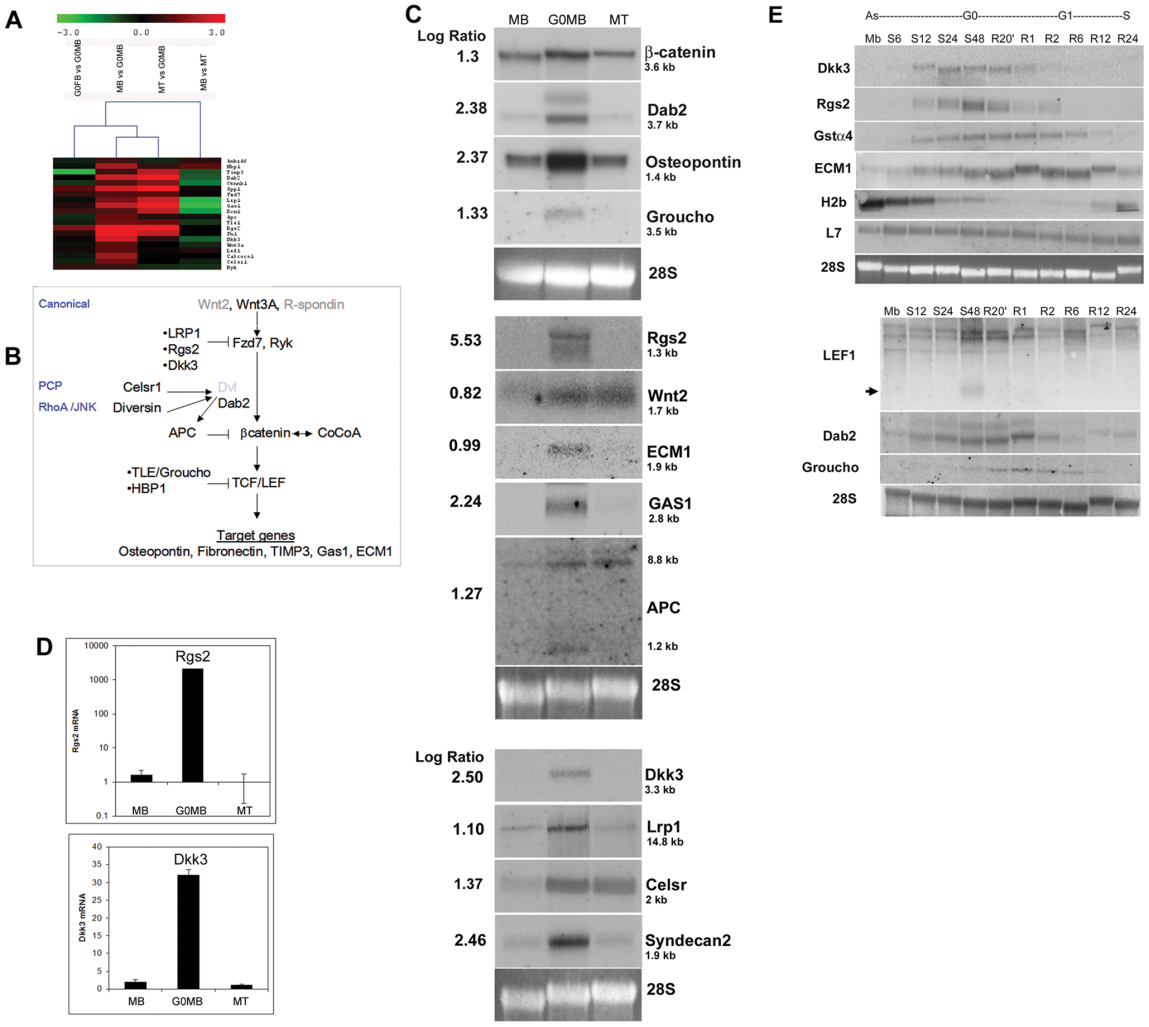
Wnt pathway genes are enriched in quiescent myoblasts. (A) Microarray analysis identifies a Wnt signature in quiescence. Cluster analysis of the 4 sample pairs analysed in Fig. 2 reveals induction of Wnt pathway genes in G_0_ MB-many of these genes are also induced in G_0_ FB but not in MT, indicating greater transcriptomal relatedness between the quiescent state of different cell types (G_0_ FB vs G_0_ MB) than between reversibly and irreversibly arrested cells of the same cell type (G_0_ MB vs MT). (B) Schematic of Wnt signaling depicting genes induced in G_0_ Mb. Genes identified as enriched in G_0_ by microarray analysis are shown in black, Wnt2 and R-spondin whose expression was directly tested are in grey. Dvl, an important Wnt signaling node is depicted for clarity (was not recovered in array). Note that components of both canonical and planar cell polarity (PCP) pathways were induced as well as some components that cross-talk with Rho/Jnk pathways. (**C**) Northern blot analysis of selected Wnt-related genes on RNA isolated from proliferating (MB), quiescent (G_0_ MB) and differentiated cells (MT). Numbers to the left of blots represent log ratios derived from comparison of G_0_ MB to asynchronous MB. Gene symbols and mRNA sizes are shown on the right. All genes tested in this independent assay show expression patterns that support their recovery in the microarray experiment. (**D**) Q-RT-PCR analysis of two putative Wnt pathway genes Rgs2 (top graph) and Dkk3 (bottom graph) in proliferating (A), quiescent (G_0_) and differentiated (MT) cells. Values represent normalized fold differences between GAPDH (control) mRNA and Rgs2/Dkk3 mRNAs in each sample calculated from cycle thresholds [2^−(−ΔΔCt)^] (n = 3, p<0.05). (**E**) Wnt pathway genes are rapidly induced in G_0_, rapidly suppressed during G1. Northern analysis of selected Wnt pathway genes during a time course of entry and exit from quiescence. Asynchronous MB (MB), cells in suspension culture for 6, 12, 24 and 48 hrs (S6, S12, S24, S48) or reactivated into the cell cycle for 20 min to 24 hrs (R20’, R1–R24). The suspended population is completely arrested by 48 hours (S48); thus ‘S48’ time point corresponds to ‘G_0_ MB’ depicted in all other figures. S-phase specific (replication-dependent) histone H2B expression is suppressed as cells arrest in suspension culture, reactivated at 24 hrs after replating when cells re-enter DNA synthesis. L7 and 28S are loading controls. Wnt pathway regulatory genes- LEF1, Groucho, Dab2 as well as putative Wnt pathway genes Dkk3, Rgs2 are more tightly quiescence-dependent (expression lost by 2–6 hrs after reactivation) than Wnt target gene Ecm1 (expression down-regulated but still detected at 24 hrs after reactivation). Arrow shows a smaller LEF1 transcript seen only in G_0_.

The most striking and surprising signature induced in G_0_ MB was a cohort of 21 genes involved in Wnt signaling (Table 1C, Fig. 3). These genes were not induced during differentiation (Fig. 3A), suggesting a quiescence-specific role. Further, though several were in common with G_0_ FB, distinct Wnt regulators were induced in G_0_ MB (Fig. 3A). The Wnt pathway is a critical regulatory module implicated in tumorigenesis, and developmental processes including self-renewal and differentiation [39] [40]. Wnt signaling is well documented in somites [41] [42] but its role in post-natal myogenesis and regeneration is still being uncovered [34] [43]. Although often associated with proliferation, Wnt signaling plays cell-type and cell-context dependent functions [44], [45].

Of the Wnt pathway genes, Wnt3A, and key positive components (Wnt receptor Fz7, transcription factors β-catenin and TCF/LEF1) were enriched in G_0_ (Fig. 3B). A larger number of negative components (APC, GSK3b, Dab2, TLE, HBP1, Lrp1, LrpAP1 (CD91) and putative Wnt inhibitors Rgs2, Dkk3) were also induced in G_0_, suggesting activation of Wnt negative feedback signaling [46] [47] [48]. Importantly, several direct/indirect Wnt targets (fibronectin, osteopontin, Gas1, Ecm1, TIMP3) were induced in G_0_, suggesting a possible functional activation.

### Validation of Wnt Pathway Gene Expression in G_0_

**Figure 4:**
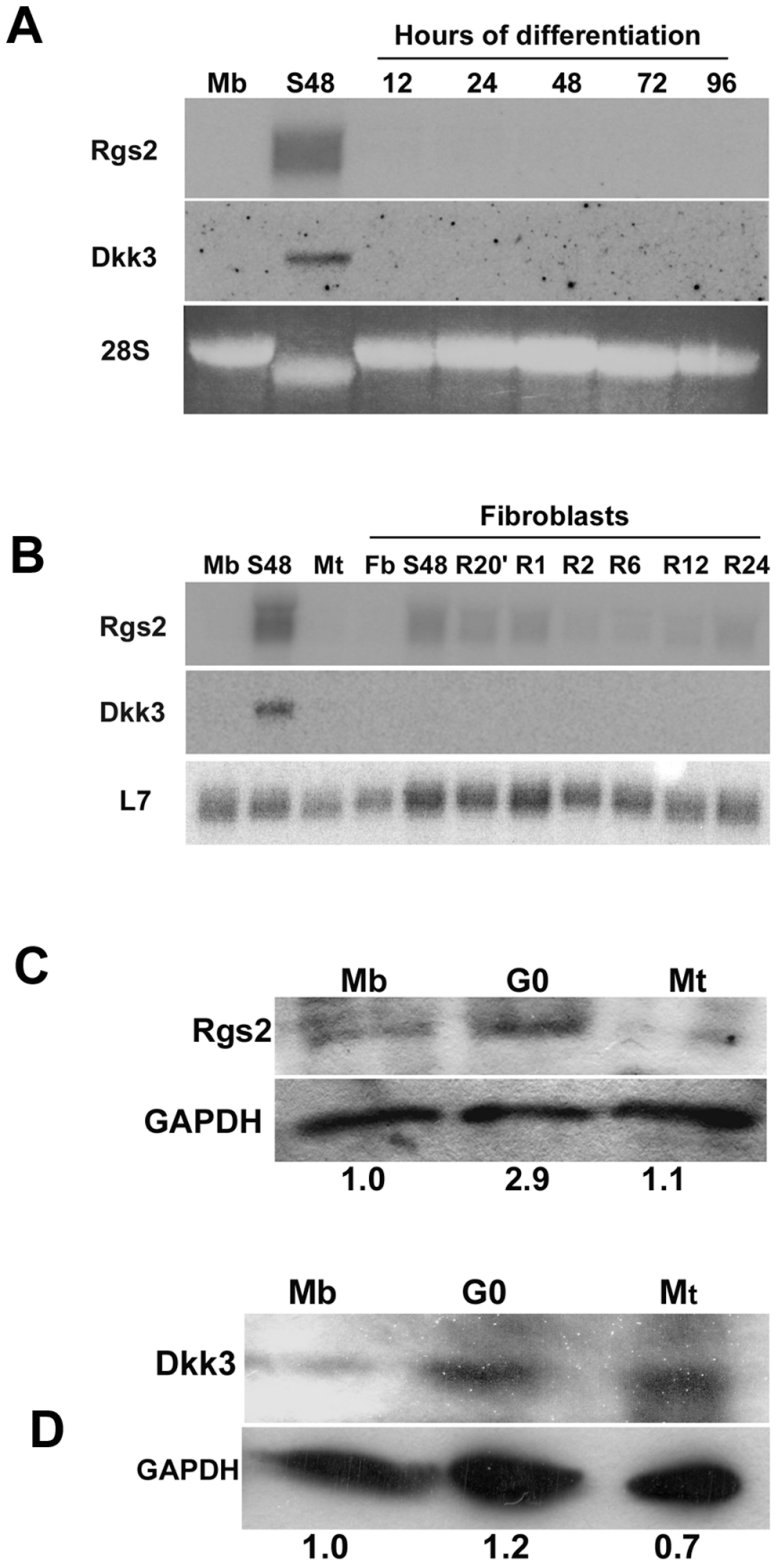
Expression profile of Rgs2 and Dkk3 in different cell states. (**A**) Quiescence-induced genes Rgs2 and Dkk3 are not expressed throughout myogenic differentiation, confirming the distinct induction pattern in reversible arrest and not in irreversible arrest. Northern analysis of growing MB (Mb) shifted to DM for 12–96 hrs. G_0_ Mb are shown for comparison. (**B**) Quiescence-induced expression of Rgs2 and Dkk3 is cell-type specific – these putative Wnt pathway genes are differentially expressed in MB and FB. Northern analysis of growing C3H10T1/2 FB (Fb), suspension-arrested FB (S48) and G_0_ FB reactivated for 20 minutes to 24 hrs. Myoblast samples are shown for comparison. Rgs2 transcript is mildly induced when C3H10T1/2 FB exit from proliferation into G_0_ [compare with strong G_0_ induction in C2C12], but Dkk3 mRNA is not detected in FB, suggesting muscle-specific activation restricted to the quiescent state. (C,D) Induction of Rgs2 and Dkk3 protein expression in quiescent myoblasts. Western blot analysis of total protein isolated from growing (MB), quiescent (G_0_) and differentiated (MT) C2C12 muscle cells and probed with antibodies against Rgs2 (C) and Dkk3 (D). In both cases the induction of protein levels is modest compared to the strong induction of the respective transcripts, potentially reflecting the strong translational repression typical of G_0_. Data depicted are representative of 3 independent experiments.

Expression of several Wnt regulators (APC, β-catenin, LEF, Groucho, Wnt2, Dab2, Lrp1, Celsr, Syndecan2, putative regulators Rgs2, Dkk3) and target genes (Osteopontin, ECM1, Gas1) was confirmed by Northern blotting (Fig. 3C, E) -most transcripts were ∼ 2–4 fold enriched in G_0_, but putative inhibitors Dkk3 and Rgs2 were more strongly induced (∼30 and∼1000 fold detected by Q-PCR (Fig. 3D). To further define the expression of Wnt components, we used time courses of arrest and differentiation. Rgs2 and Dkk3 transcripts showed gradual induction during G_0_ entry, and rapid suppression upon cell cycle re-entry, consistent with an incompatibility with progression (Fig. 3E). Neither Rgs2 nor Dkk3 was activated during differentiation (Fig. 4A). We also probed the differential induction in quiescent myobasts vs. quiescent fibroblasts. Rgs2 was faintly induced when fibroblasts entered quiescence and down-regulated during reactivation as in myoblasts (Fig. 4B), Dkk3 was not detected in G_0_ FB nor at any time during reactivation. These results confirm quiescence-specificity of Rgs2 and Dkk3 transcript induction as well as a component of muscle-specific regulation. Rgs2 and Dkk3 protein levels were also induced in G_0_, (Fig. 4C, D), albeit modestly in comparison to the strong rise at the mRNA level.

To comprehensively analyze the expression of the Wnt pathway, a targeted survey of Wnt components was performed using a QPCR ‘superarray’ of 84 Wnt pathway genes, revealing distinct differences between these 3 cellular states with respect to Wnt components (Table S3). Interestingly, transcripts encoding Wnt ligands [Wnt4 (>600 fold), Wnt16 (>80 fold)], as well as Wnt antagonists [Frzb (>800 fold induced in G_0_), sFRP4 (>200 fold)] were strongly upregulated, confirming induction of the Wnt pathway in G_0_. Taken together, this analysis establishes that the Wnt pathway shows altered expression in different cellular states and defines a characteristic set of Wnt pathway components expressed in quiescent myoblasts.

### Functional Analysis of Canonical Wnt Signaling Reveals differences between G_0_ Myoblasts and Myotubes

**Figure 5:**
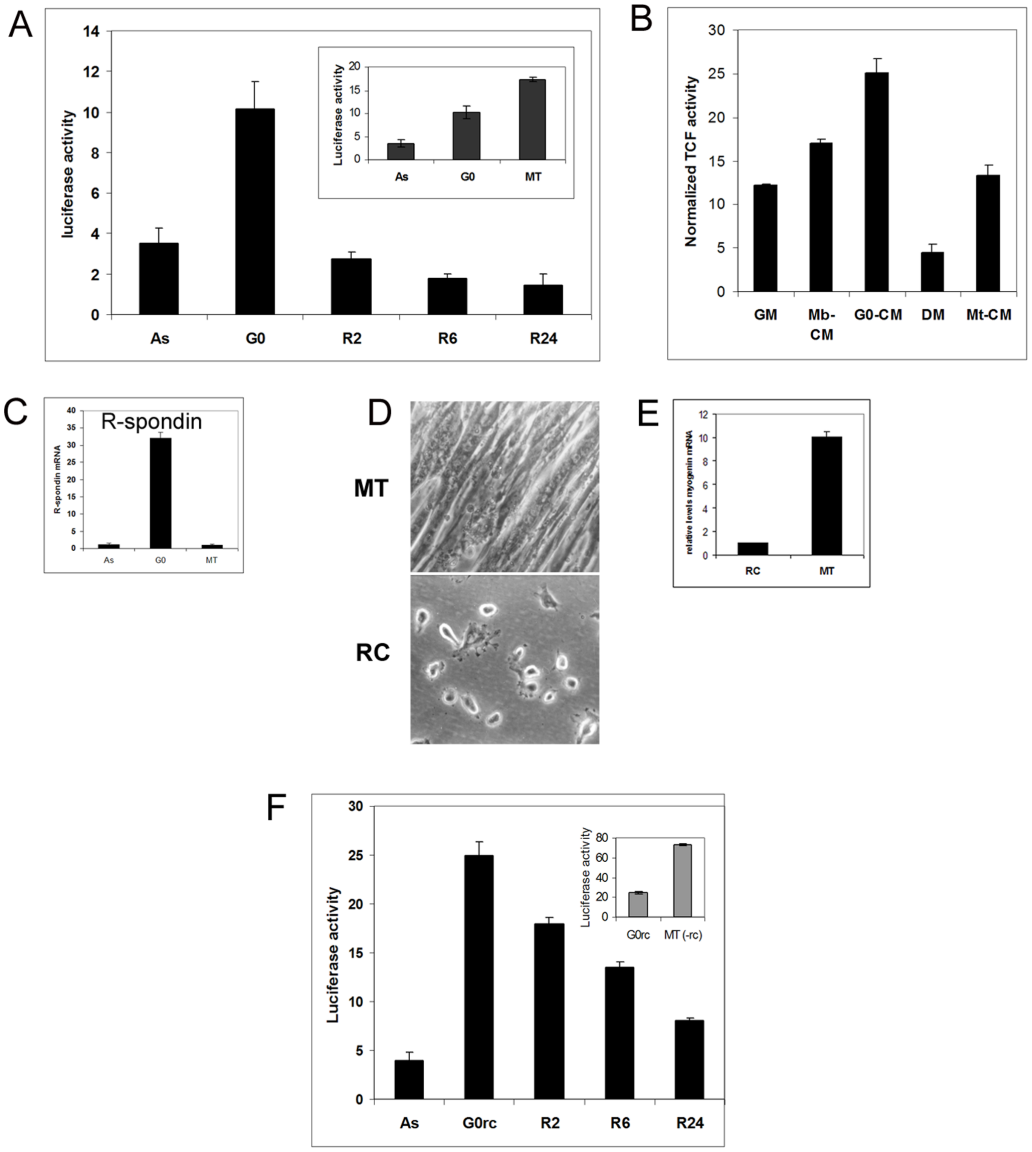
Moderate induction of Wnt signaling in two models of quiescence. (**A**) Wnt signaling revealed by TCF-dependent luciferase activity in stabily transfected TOPflash myoblast clone TFC1. Asynchronously proliferating myoblasts (As), suspension-arrested quiescent myoblasts (G_0_), suspension-synchronized myoblasts after re-activatation for 2–24 hrs (R2–R24), or myoblasts induced to differentiate for 72 hrs (MT). Transcriptional activity of the Wnt responsive TCF reporter is induced as MB enter quiescence and rapidly suppressed upon re-activation into the cell cycle. TCF-dependent luciferase activity (rlu/µg protein) is moderately induced in G_0_ arrest but strongly induced in differentiation-associated arrest (MT, inset). Data represents mean +SE from atleast 3 independent experiments. (**B**) Conditioned medium (CM) from G_0_ MB cultures contains more Wnt/TCF reporter-inducing activity than CM from proliferating or differentiated cultures. TOPflash reporter cells were exposed to CM derived from G_0_ cultures (G_0_-CM), or proliferating cultures (Mb-CM), or differentiated cultures (Mt-CM). Fresh growth medium (GM) and differentiation medium (DM) were used as controls. Data represents mean +SE from 3 independent experiments. (**C**) Secreted Wnt agonist R-spondin expression is strongly induced in G_0_. RNA isolated from growing (Mb), arrested (G_0_) and differentiated (Mt) muscle cells was analysed by Q-RT-PCR (n = 3). (**D–F**) Wnt signaling is induced in an independent culture model of G_0_ MB (reserve cells). (D) Phase contrast photographs of quiescent mononucleated undifferentiated reserve cells (RC) after differential trypsinization specifically removed myotubes (MT) in 5-day differentiated TFC1cultures. (E) RC isolated from away from MT do not induce MyoG (QRT-PCR analysis), confirming their undifferentiated state. (F) TOPflash TCF reporter activity is induced in purified quiescent reserve cells (G_0_rc) and its decline in reserve cells that have been reactivated by the addition of GM for 2–24 hrs (R2–R24). Inset shows TOPflash activity in G_0_rc compared to purified MT cultures depleted of reserve cells [MT(-rc)]. Data represents mean +SE from 3 independent experiments.

In G_0_ MB, the level of Wnt signaling could not be predicted from expression patterns, since many direct targets (fibronectin, TIMP3) were up-regulated but others (cyclin D1, c-myc) were suppressed. To functionally examine canonical Wnt signaling in G_0_ MB, we generated a stable clone of C2C12 cells (TFC1) expressing TOPflash, a reporter of TCF/LEF transcriptional activity [49]. Compared to proliferating myoblasts, Wnt reporter activity was strongly induced in MT (∼6 fold), but moderately induced (∼3 fold) in G_0_ (Fig. 5A). Interestingly, the induction of TCF activity in quiescence was reversed during re-activation into the cell cycle. Thus, strong Wnt signaling is associated with irreversible arrest, but importantly, reversible arrest was associated with reversible induction of Wnt signaling.

### Secreted Wnt Activity is Increased in G_0_

To determine whether more Wnt signaling activity is secreted in G_0_, we examined the effect of conditioned media (CM) from equal numbers of MB, G_0_ MB or MT derived from the parental C2C12 line, on TCF transcriptional activity in the TFC1 reporter cells. CM from MT elicited low TCF activity in reporter cells, by contrast to strong activation of TOPflash within MT. However, CM from G_0_ MB elicited higher Wnt reporter activity than CM from either MB or MT (Fig. 5B).

To examine whether the disparity in secreted Wnt signals between G**_0_** MB and MT might be due to other secreted Wnt agonists such as R-spondin, which activate signaling via Frizzled binding [50], we used QPCR. R-spondin transcripts were induced 32-fold in G_0_ but not in MT (Fig. 5C), and might contribute to Wnt pathway activation despite the elevated levels of antagonists (Frzb, sFRP4).

### Intermediate, Reversible Wnt Signaling Activation in Another Quiescence Model

β-catenin, the transcriptional effector of Wnt signaling is also associated with cadherin-rich cell adhesion complexes. As detachment of cells from the substratum could cause redistribution of β-catenin, potentially increasing its nuclear availability, we considered the possibility that the suspension-induced rise of TOPflash reporter activity may reflect disruption of adhesion complexes rather than entry into G_0_. To test this hypothesis, we employed an attached cell model of quiescence. Reserve cells (RC) are a minor population that arises during mitogen withdrawal-induced differentiation. These cells can constitute 10–30% of a myotube culture, and resist fusion and differentiation. Like suspension-arrested MB, RC remain mononucleated, enter G_0_ and express markers of resting SCs such as Pax7 and CD34 [16]. Although not well understood, determination of RC is thought to involve Wnt and insulin signaling [51]. To further explore Wnt signaling in G_0_, we generated RC from TFC1 Wnt reporter cells (Fig. 5D). MyoD (not shown) and MyoG were not expressed in RC but strongly induced in MT (Fig. 5E), validating the cell enrichment procedure. As in suspension-arrested G_0_ MB, TCF activity was moderately induced in G_0_ RC and down-regulated during their re-activation (Fig. 5F).

The reversible induction of TCF activity in both these quiescence models (suspension-arrested MB, attached reserve cells) suggests that Wnt signaling plays a role in G_0_ irrespective of the pathway of G_0_ entry, and is not merely the consequence of cell detachment. Since TCF induction is moderate in G_0_ but strong in MT, either levels of Wnt activation or co-operation with different signaling pathways may distinguish G_0_ from irreversible arrest. Moderate Wnt activation also appears to be important for reprogramming of somatic cells to iPS cells, while strong activation is inhibitory [52].

Taken together, these results using a transfected reporter show that the distinct and reversible Wnt expression pattern observed in quiescent myoblasts correlates with functional changes in canonical Wnt signaling that are reversed on cell cycle entry.

### Differential β-catenin Occupancy at Myogenic Promoters in G_0_ vs. MT

**Figure 6:**
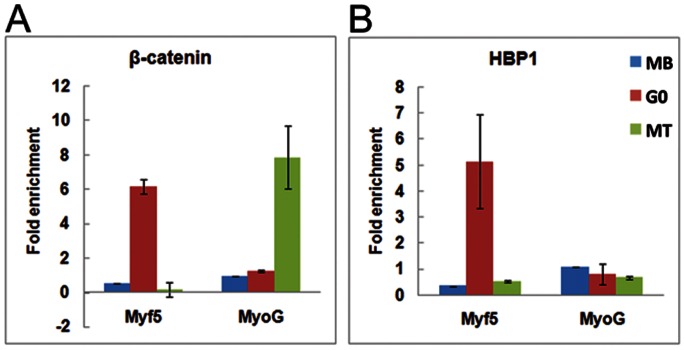
Chromatin-IP analysis of Myf5 and MyoG promoters in different cellular states. Antibodies against β-cat or HBP were used to assess the association of these Wnt-regulated transcription factors with chromatin in different cellular states as described in Materials and Methods. Control pulldowns used IgG and all values shown represent fold enrichment of the specific transcription factor after normalization against control IgG values. (**A**)**.** The Wnt target transcription factor β-cat associates with the known Wnt-responsive site in Myf5 enhancer preferentially in G_0_ (G_0_) but shows low enrichment in either proliferating (MB) or differentiating muscle cells (MT) (Blue bars-MB; pink bars-G_0_; green bars-MT). This observation is consistent with the hypothesis that Wnt signaling is active in quiescent myoblasts. Comparison of β-cat association on another myogenic promoter (Myogenin promoter) shows greater enrichment in MT. Taken together, these observations suggest that Wnt/β-cat regulates different genes in different cellular states. (**B**)**.** ChIP analysis shows that HBP1 (a Wnt-induced repressor) co-associates with the Myf5 enhancer only in G_0_ (Blue bars-Mb; pink bars-G_0_; green bars-MT) and does not associate with this element in either proliferating or differentiated muscle cells. This observation suggests that fine-tuning of Myf5 expression by both activating and repressive mechanisms may occur in quiescent cells by association of two types of Wnt-responsive transcription factors. Taken together, this observation would account for the absence of induction of Myf5 mRNA in quiescent myoblasts despite the association of the transcriptional activator β-cat.

To explore the role of Wnt signaling in regulating muscle genes during reversible and irreversible arrest, we assessed occupancy of the canonical Wnt target transcription factor β-cat on endogenous myogenic promoters. Expression of Myf5, a known transcriptional target of β-cat [35], is associated with self-renewal and lineage determination and down-regulated upon overt differentiation. Myogenin (MyoG), a differentiation-inducing factor is not known to be a direct target of β-cat. Ch-IP analysis revealed that β-cat is differentially associated with Myf5 and MyoG regulatory sequences in different cellular states (Fig. 6A). In MT, where MyoG but not Myf5 is strongly expressed, β-cat was associated with chromatin at the MyoG promoter but not the Myf5 enhancer. In G_0_, where neither Myf5 nor MyoG is expressed, β-cat was not found at the MyoG promoter but surprisingly, was enriched at the Myf5 enhancer. Reasoning that absence of Myf5 expression under these activating/permissive conditions might be blocked by repressive factors induced in G_0_, we assessed the occupancy of a known Wnt inhibitor HBP1 (Fig. 6B). Indeed, HBP1, a repressor that directly competes with Tcf/Lef on Wnt-responsive promoters, and which was up-regulated in G_0_ (Table 1), associates with the repressed Myf5 enhancer in G_0_, but not with the active MyoG promoter in MT. Thus, expression of myogenic genes in G_0_ vs. MT is associated with distinct combinations of Wnt-responsive transcriptional activators and repressors, and HBP1, a known myogenic and Wnt inhibitor, [53] may fine-tune regulation of muscle-specific genes in quiescence vs. differentiation.

### Enhancing Wnt Signaling in Suspension Culture Subverts the Quiescence Program

**Figure 7:**
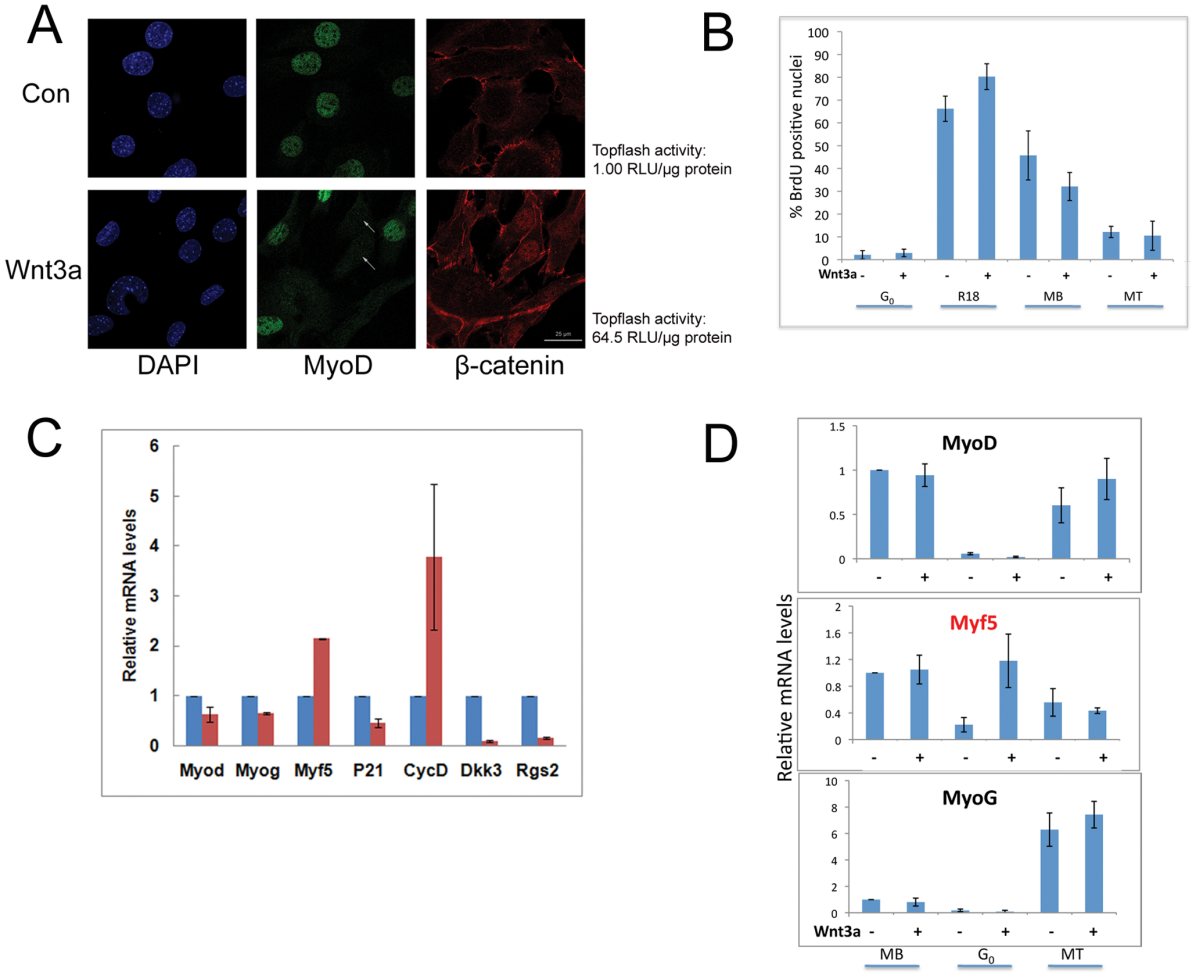
Enhancement of Wnt signaling subverts the quiescence program. (A) Exposure of adherent MB to rWnt3a (50 ng/ml) leads to β-cat nuclear localization, TOPflash activation and suppression of MyoD protein as compared to control cells. (B) rWnt 3a (50 ng/ml) does not enhance proliferation (BrdU incorporated in a 30′ pulse) in muscle cells: Asynchronous MB, G_0_ MB, MB reactivated after synchronization in (R18) or differentiated myotubes (MT) [Note: all BrdU+ nuclei in myotube cultures were in residual mono-nucleated myobalsts]. Values represent the mean±SEM from three independent experiments. (C) Exogenous Wnt3a alters the quiescence program: Q-RTPCR analysis of control (blue bars) and Wnt-treated (pink bars) cells held in suspension for 48 hrs shows repression of MyoD and MyoG but induction of Myf5, indicating differential response of MRFs; repression of p21 and induction of CyclinD1 collectively suggesting a shift to a proliferative gene expression program; and finally, repression of quiescence-induced genes Rgs2 and Dkk3, consistent with this shift. Values represent the mean±SEM from three independent experiments. (D) Context-dependent response to Wnt enhancement. Cells in three different states (MB, G_0_ or MT) were treated for 48 hours with 50ng/ml of rWnt3a. Of the MRFs, Myf5 mRNA is only induced by Wnt3a if the target cells are in G_0_. Values represent the mean±SEM from three independent experiments.

The results thus far indicate that reversible arrest in myoblasts is defined by a distinct transcriptional program, of which the Wnt pathway comprises a quiescence-induced module. Further, the differential elevation of TCF/β-cat transcriptional response in MT (strong) vs. G_0_ (moderate) suggested that the level of Wnt signaling maybe important for achieving these distinct out-of-cycle states. Conceivably, moderate Wnt activation may be functionally linked with achieving arrest in an undifferentiated state, while strong Wnt activation may promote differentiation-coupled arrest. To test this model, we enhanced Wnt signaling in conditions that normally induce G_0_, by treating suspension cultures with rWnt3a. rWnt3a was active as evidenced by induction of β-catenin translocation and TOPflash activity, both known consequences of Wnt signaling and also repressed MyoD protein (Fig. 7A). Unlike the well-documented proliferative response reported in other cell types [54] [55], rWnt3a did not activate proliferation of G_0_-arrested cells (Fig. 7B). Analysis of MRF expression showed that Myf5 mRNA was induced by exogenous Wnt treatment of G_0_ cells (Fig. 7C) consistent with its status as a direct Wnt target. However, MyoD and MyoG mRNAs were not induced, suggesting that strong Wnt signaling is not sufficient to re-direct G_0_ towards differentiation. To test whether initial cell state is important for interpreting the Wnt signal, we added rWnt3a to proliferating, arrested or differentiated cells. Interestingly, Myf5 mRNA was induced only if target cells were already in G_0_- neither MB nor MT up-regulated Myf5 in response to rWnt3a (Fig. 7D), suggesting that cellular context is important for Wnt signaling. Since many Wnt components are specifically induced in G_0_, this data suggests quiescence-dependent signal responsiveness.

Further, β-cat (also induced in G_0_) is associated with Myf5 enhancers in quiescent but not in proliferating or differentiated cells (Fig. 6), consistent with the quiescence-specific induction of Myf-5 transcript by exogenously added Wnt.

We further probed the Wnt response in G_0_ by examining other genes. Firstly, the cell cycle inhibitor p21 was repressed and the pro-proliferative CyclinD1 was induced (Fig. 7C), but DNA synthesis was not activated (Fig. 7B), suggesting that while proliferation-promoting changes might be induced by Wnt3a, these were not sufficient to completely reverse arrest in non-adherent cells. Secondly, expression of two strongly quiescence-induced genes Rgs2 and Dkk3 were strongly suppressed by rWnt3a treatment. Thirdly, we employed hierarchical clustering of the QPCR “superarray” data (Table S3) to determine the degree to which Wnt treatment of G_0_ myoblasts alters the Wnt network itself. rWnt3a treatment of quiescent myoblasts drastically modified the transcriptional profile of the Wnt module by suppressing quiescence-induced components (Fig. S4). Taken together, these results suggest that moderate Wnt signaling and associated Wnt component expression in G_0_ may facilitate the induction/maintenance of quiescence. While elevating Wnt in G_0_ subverts an actively quiescence-induced gene expression program, it is not sufficient to signal a return to active proliferation.

### Strong Activation of Wnt Pathway in G_0_ Reduces Clonogenic Potential

**Figure 8:**
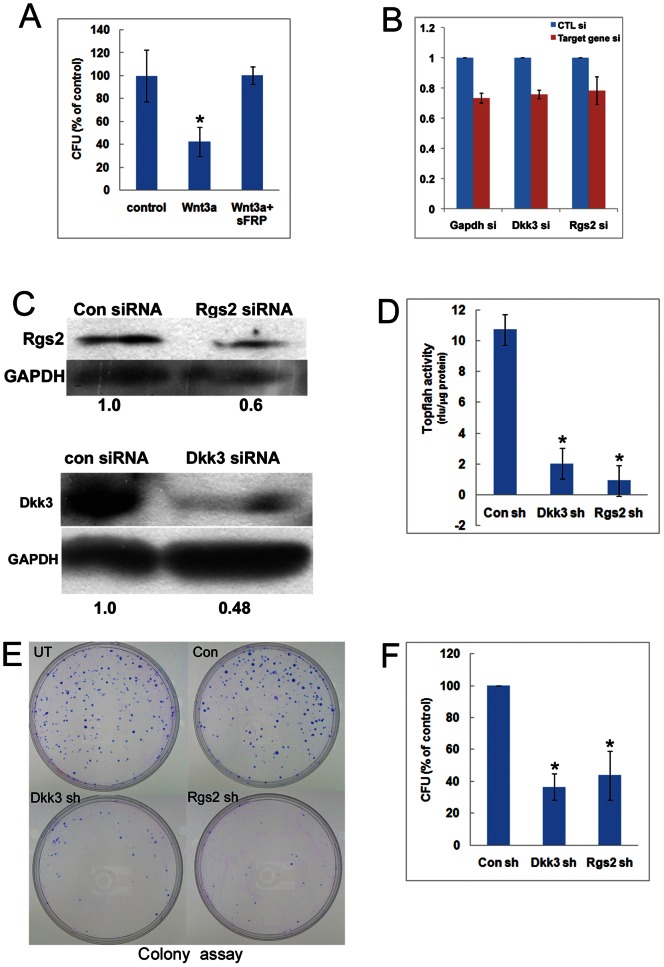
Exogenous Wnt treatment compromises a G0-induced program that promotes clonogenic potential. (A) Wnt3A treatment of MB reduces clonogenic potential. Colony formation was measured after 48 hrs in control culture conditions (either in proliferating conditions-Mb, or in suspension culture-G_0_), or in the presence of 50 ng/ml of rWnt3A. Cloning efficiency (a measure of self-renewal) was strongly reduced by Wnt3A supplementation and restored by simultaneous addition of 50ng/ml sFRP2. Values represent the mean±SEM from three independent experiments, *p*<0.05 (denoted by asterisk *). (B) Knockdown of Rgs2 and Dkk3 transcripts using siRNAs. siRNAs were designed against the putative Wnt regulators Rgs2, Dkk3 or an irrelevant gene (GAPDH) or a control scrambled siRNA sequence and transfected into C2C12 myoblasts along with a GFP plasmid. GFP**^+^** transfected cells were enriched by FACS, RNA isolated and analysed by Q-RT-PCR and the relative mRNA levels calculated. In each pair, the mRNA level is depicted of cells transfected with scrambled siRNA (blue bars) and cells transfected with the targeting siRNA (pink bars). Values represent the mean and SEM of 3 independent experiments. In each case, modest but reproducible reduction of the target transcript level is observed. (C) Reduction of Rgs2 and Dkk3 protein expression by siRNA-mediated knockdown. Western blot analysis of total protein isolated from control and knockdown C2C12 muscle cells probed with antibodies against Rgs2 (top) and Dkk3 (bottom). GAPDH protein levels indicate equal loading. Data depicted is representative of 3 independent experiments. (D) Rgs2 and Dkk3 expression is necessary for Wnt signaling. Knockdown of either Rgs2 or Dkk3 in growing or quiescent MB leads to suppression of TOPflash activity. Cells were treated and enriched as described in (B) and luciferase activity measured. Despite modest reduction of protein levels, strong reduction in TOPflash activity are seen, indicating a critical role for Rgs2 and Dkk3 in Wnt-βcat signaling. Values represent the mean and SEM of 3 independent experiments. (E,F) Knockdown cells (‘Rgs sh’ and ‘Dkk sh’) were enriched as described in (B) cultured in quiescence-inducing conditions, recovered from suspension culture and plated at clonogenic density for assessment of self-renewal (colony formation). Controls include untransfected cells (‘UT’) and control shRNA transfected cells (‘Con sh’). Typical plates with colony assays are shown in (E) and data are quantified as CFU (colony forming units) in (F). Values represent the mean and SEM of 3 independent experiments.

To test the functional consequences of exogenously enhanced Wnt signaling during the induction of G_0_, we directly assessed cloning efficiency, a measure of self-renewal. Cells treated with 50 ng/ml rWnt3A (a dose selected on the basis of Topflash activation, Fig. S5) in adherent or suspension culture showed reduced colony formation (Fig. 8A); inclusion of Wnt inhibitor sFRP2 completely reversed this negative effect, establishing the specificity of the response to Wnt. rWnt3a treatment in G_0_ did not increase either senescence (measured by senescence associated-βgal activity) nor cell death (measured by Annexin V/PI staining and FACS analysis) (Fig. S3). Thus, the self-renewal capacity associated with the modest rise in Wnt signaling normally seen during quiescence is negated by enhanced Wnt activation. Taken together, these results suggest that differences observed in the endogenous level of Wnt signaling may contribute to the distinction between reversible and irreversible arrest. Loss of clonogenicity in cells treated with Wnt may be viewed as the consequence of the altered transcriptional profile of Wnt components, which could lead to signaling conflicts that prevent cells from proliferation.

### Quiescence-induced Genes RGS2 and DKK3 are Required for Wnt Signaling and Promote Self-renewal

The strong induction of Rgs2 and Dkk3 specifically in G_0_ MB but not MT, and their rapid suppression as G_0_ cells entered G_1_ (Fig. 4E) is consistent with a role in the quiescence program. Expression of both Rgs2 and Dkk3 was suppressed when quiescent myoblasts were exposed to rWnt3a (Fig. 7C), coincident with loss of clonogenicity, and suggests a role in self-renewal. Rgs2 regulates heterotrimeric G protein (Gα) signaling in cardiac cells, but in Xenopus, ectopically expressed hRgs2 phenocopies dominant-negative XWnt8 [46], suggesting Wnt inhibitory functions. Dkk3 is related to known Wnt antagonist Dkk1, but is not thought to inhibit Wnt [48], and may even promote Wnt signaling [47]. However, the function of these genes in myoblasts was unknown. To test Rgs2 and Dkk3 function in quiescent MB, we assessed their potential role in canonical Wnt signaling. Knockdown of either Rgs2 or Dkk3 at RNA and protein level (Fig. 8B,C) suppressed canonical Wnt signaling, as evidenced by reduced TOPflash activity (Fig. 8D). Thus, in quiescent MB, rather than inhibiting the Wnt pathway, both Rgs2 and Dkk3 appear to be required for Wnt signaling.

Artificially elevated Wnt signaling in G_0_ led to loss of Rgs2/Dkk3 expression as well as reduced cloning efficiency. To investigate a possible causal relationship between Rgs2/Dkk3 expression and clonogenic potential, we used RNAi. Myoblasts in which either Rgs2 or Dkk3 were knocked down were FACS-enriched (using a co-transfected GFP plasmid), cultured in suspension for 48 h to induce G_0_, and analyzed by CFU assay. Colony formation in both Rgs2 and Dkk3 knockdown cells was reduced compared to control transfected or untransfected cells (Fig. 8E,F), supporting the hypothesis that these quiescence-induced, Wnt regulatory genes play a critical role in maintaining self-renewal potential in G_0_.

Collectively, these studies demonstrate that the Wnt signature identified as enriched in G_0_ myoblasts by unbiased profiling has functional consequences for quiescence, and identify two new regulators of the quiescence program, Rgs2 and Dkk3, both of which are required for canonical Wnt signaling in quiescent cells.

### Summary and Conclusions

The control of reversible quiescence, a cellular state with important implications for stem cell function and tumor biology is incompletely defined. A core quiescence program has been described in lymphocytes, HSC and fibroblasts [2] [4] [5], but no genome-wide analysis has compared reversible arrest to other physiological, viable out-of-cycle states. Several important concepts emerge from our analysis. Firstly, distinct genetic programs control reversible and irreversible [differentiation-associated] arrest. A key component of the reversible quiescence program in myoblasts is an active suppression of differentiation by multiple failsafe mechanisms. This muscle inhibitory program is also induced in G_0_ fibroblasts, and could account for their ability to resist differentiation by ectopic MyoD [2] [3]. Secondly, tissue-specific features overlay the core quiescence program. Although G_0_ MB and G_0_ FB share a common transcriptional profile, a significant number of unique genes are enriched in each cell type alone. Thirdly, a distinct constellation of tumor suppressors/cell cycle inhibitors is induced in reversible arrest, which in combination with the large number of inhibitors of differentiation emphasizes distinct strategies for achieving and maintaining the quiescent state. Fourthly, a number of genes are conserved between cultured G_0_ MB and freshly isolated SC, which may reflect conserved strategies for survival/self-renewal. Finally, Wnt signaling, a key regulator of stem cell self renewal regulates the induction/maintenance of the quiescent state. The surprising association of Wnt signaling with reversible arrest is discussed in detail below.

### Unexpected Association of Wnt Signaling with the Quiescent State

Wnt signaling is most commonly associated with proliferation and its deregulation is clearly implicated in tumorigenesis [44]. However, growing evidence suggests context- and cell type-dependent interpretation of Wnt signals [44], [56]with outcomes other than proliferation (such as differentiation, apoptosis [43]). In addition to the intrinsic complexity of the Wnt pathway (multiple Wnt ligands, receptors, co-receptors, co-inhibitors and target transcription factors), there are synergistic and antagonistic interactions with other signaling pathways. Viewed against this complexity, the distinct state-specific outcomes we report in the myogenic cell system provide an opportunity to distinguish context-dependent sub-networks.

Three observations support the view that the distinct Wnt pathway transcriptional signature in quiescent MB reflects functional Wnt signaling. First, canonical Wnt pathway reporter activity showed a moderate rise in G_0_, reaching a level intermediate between levels in proliferating and differentiated muscle cells. The intermediate level appears to be important since enhancing Wnt levels in G_0_ myoblasts by addition of exogenous rWnt3a protein not only altered expression patterns typical of G_0_ myoblasts [myogenic factors, quiescence-induced genes, cell cycle genes, Wnt genes], but also had deleterious effects on clonogenicity. These observations suggest that signaling through the endogenous Wnt pathway is regulated and moderate induction provides a specific benefit to quiescent cells. Second, two genes most highly induced in G_0_ (Rgs2 and Dkk3) were found to be required for canonical Wnt signaling. Third, altering expression of these Wnt pathway activators (Rgs2 and Dkk3) negatively affected clonogenic survival of G_0_ MB. Taken together, these results strongly support a functional role for moderate activation of the Wnt pathway in attaining/maintaining quiescence. This leads us to speculate that a threshold level of Wnt signaling may be essential for survival in G_0_, but over-shooting that level is restrictive.

### Wnt Signaling, Survival and Self-renewal

The context-dependent role of Wnt in muscle cells also suggests that other factors (such as IGFs) may co-ordinately induce differentiation. Wnt and insulin have synergistic effects on muscle differentiation and hypertrophy [51], and in G_0_ MB, lower expression of IGFs, IGFR and IGF-BP than in MT may contribute to their undifferentiated state. Loss of MyoD expression when nuclear β-cat expression is induced would also reinforce the undifferentiated state.

An important function of Wnt signaling may be to promote cell survival [54], through IGFs, [57] and PI3 kinase/Akt [58] [59]. Thus, in G_0_ MB, moderate Wnt activation and low levels of IGFs may combine to protect cells against apoptosis and aid in cell survival by activating the PI3kinase/Akt pathway. Our finding that colony formation is adversely affected when Wnt signaling is enhanced supports this notion.

Wnt signaling has been implicated in self-renewal of epithelial stem cells as well as ESCs and HSCs [40] [60]. Since G_0_ MB in culture share many features of muscle SCs in vivo, we hypothesize that moderate Wnt signaling in quiescent SCs may combine with other factors in the niche (e.g. Notch) to promote survival and self-renewal and inhibit differentiation. A mild increase in Wnt signaling in aged mice leads to suppression of SC proliferation and conversion to a non-myogenic fate [43]. Further, enhancement of Wnt signaling in the bone marrow niche compromises quiescence and self-renewal of HSCs [55]. Finally, different Wnts show distinct effects on SC proliferation while Wnts 1,3 and 5 are stimulatory, Wnt 4 and 6 are inhibitory [34]. Canonical Wnt signaling is context dependent: both the magnitude of the signal as well as the combinatorial involvement of modifiers/other signals may decide the balance of β-cat’s interactions and cellular fate. Our finding that Myf5 mRNA expression is absent in G_0_ myoblasts despite the presence of β-cat at the Myf5 enhancer, but consistent with the combined presence of the repressor HBP1 supports this notion.

Overall, our results demonstrate that far from being a passive state into which cells regress, quiescence is an actively regulated state associated with the induction of a distinct transcriptional program. This quiescence program is evolutionarily ancient and signal-dependent, and has common as well as tissue-specific features. The induction of a quiescence program may be central to stem cell maintenance by precluding entry into other stationary states such as differentiation or senescence. Therefore, understanding the mechanisms that induce, maintain and break quiescence has implications for conditions where these programs are compromised or enhanced, contributing to cancer and degenerative disease respectively.

## Supporting Information

Figure S1 **Cell cycle gene expression in adherent and suspended myoblasts: cell cycle arrest is evident.** Gen-Mapp diagram of cell cycle gene expression derived from microarray data (normalized log ratios) comparing adherent proliferating myoblasts (Mb) with 48 hr suspension arrested myoblasts (G0 Mb). Green boxes represent genes down-regulated in G0 and red boxes represent genes upregulated in G0 (white boxes surround genes that participate in the cell cycle but were not spotted on the array). Expectedly the entire cell cycle network is suppressed coincident with the induction of genes such as the tumor suppressor p53 and the TGFb target transcription factor Smad3 (see Figure S2 for TGFb pathway induced in G0). Interestingly, the Orc4l DNA binding subunit of the Origin Replication Complex is induced, perhaps indicating a mechanism that marks origins in reversibly quiescent cells.
(TIF)

Figure S2 **The TGFb pathway is induced in G0 myoblasts.** The TGF-b signaling network is up-regulated in quiescent myoblasts. Genes positively induced include participants at all levels of the pathway including cell surface receptors, co-receptors, transcriptional effectors and target genes. Negative regulator SnoN is repressed in quiescent myoblasts, indicating an overall induction of TGFb signaling, a known participant in quiescence and repression of myogenesis.
(TIF)

Figure S3 **Wnt 3A treatment of quiescent myoblasts does not induce either apoptosis or senescence.** The negative effects of Wnt on clonogenic self-renewal (Figure 8A) were not a result of induction of cell death or senescence pathways. Myoblasts were cultured in methocel suspension for 48 hours in the absensce (G0) or in the presence (G0+ Wnt3A). Both cultures were harvested and either stained for cell surface Annexin 5 or propidium iodine (PI) and analysed by flow cytometry. Senescence associated b-galactosidase activity was detected by cytological staining using a chromogenic substrate. Wnt 3A treatment of quiescent myoblasts does not increase rates of either apoptosis or senescence.
(TIF)

Figure S4 **Wnt3A treatment of quiescent myoblasts drastically alters expression of the Wnt module.** Hierarchical clustering of Wnt super-array data reveals that Wnt3A treatment of quiescent myoblasts drastically alters expression of the Wnt module. Four clusters of genes were readily discerned: (1) Genes strongly induced specifically in G0 but not in MT, and repressed in response to Wnt 3A. (2) Genes suppressed in G0 and strongly induced by Wnt3A. (3) Genes strongly induced in MT, mildly induced in G0 (common to two states of arrest), but suppressed by Wnt3A. (4) Genes mildly induced in G0 and strongly suppressed by Wnt3A. The alteration of nearly all genes in the Wnt module by enhanced Wnt signaling suggests the operation of the Wnt feedback control mechanism.
(TIF)

Figure S5 **Dose response of Wnt3a treatment on TOPflash activity.** Stably transfected Wnt reporter myoblasts (TFC1) were treated with different doses of Wnt3A (10, 50, 100 ng/ml) and TOPflash luciferase activity measured after 48 hours. 50 ng/ml elicited nearly as strong a response as 100 and was chosen for further experiments.
(TIF)

Table S1 **Selected Genes induced in G0 myoblasts.** Based on Gene ontology searches, genes induced in quiescent myoblasts were classified into different functional classes. A partial list of the ∼1100 quiescence-induced genes is presented.
(DOC)

Table S2 **Genes commonly enriched in Quiescent C2C12 myoblasts (this study) and freshly isolated muscle satellite cells (Fukuda et al, 2007).** Based on comparison of the data generated in this study (1.6 fold up-regulated in G0) with the data generated from freshly isolated mouse SC (Fukada et al, 2007; 5-fold up-regulated in G0), a list of commonly G0-induced genes is presented. Note the common induction of SC markers CD34 and Sca1, Wnt regulator Rgs2, signaling components Stat3 and Decorin, Stem cell marker Klf4, all indicating a shared network in quiescent cells in vitro and in vivo.
(DOC)

Table S3 **Wnt pathway gene expression in proliferating myoblasts, myotubes, G0 myoblasts and G0 myoblasts treated with Wnt 3A [50 ng/ml].** Alteration of the Wnt module by treatment of G0 myoblasts with the ligand Wnt3a suggesting feedback control.
(DOC)

Materials and Methods S1 Information on antibodies, primers and si/shRNAs used in this study can be accessed in the Supporting Information Materials and Methods.
(DOC)
