# Supplementary figures and images for: Distinct Transcriptional Networks in Quiescent Myoblasts: A Role for Wnt Signaling in Reversible vs. Irreversible Arrest
Source: PLoS One. 2013 Jun 3;8(6):e65097. doi: 10.1371/journal.pone.0065097 (PMC3670900; doi:10.1371/journal.pone.0065097)

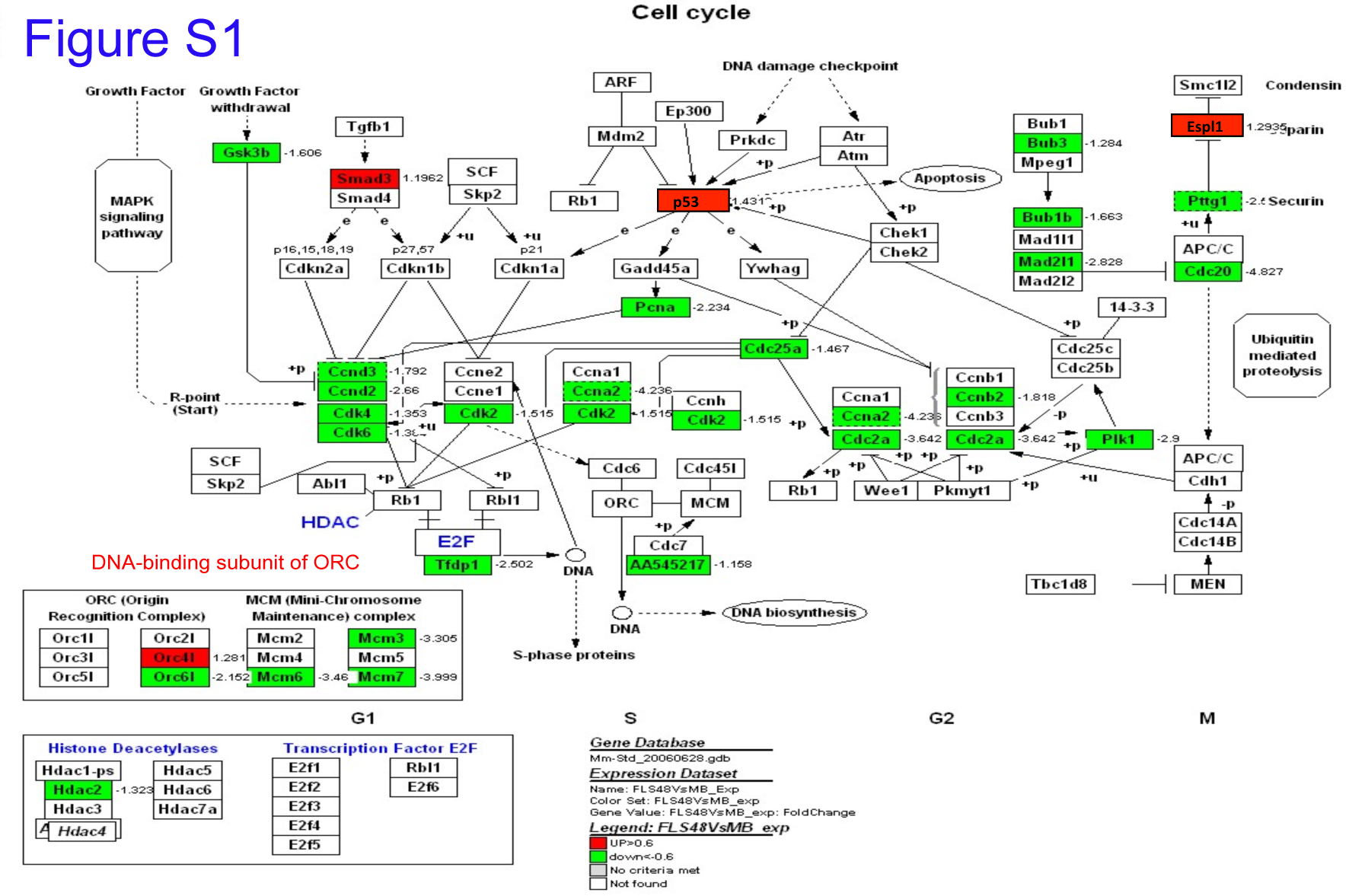

Supplement: Figure S1 — Cell cycle gene expression in adherent and suspended myoblasts: cell cycle arrest is evident. Gen-Mapp diagram of cell cycle gene expression derived from microarray data (normalized log ratios) comparing adherent proliferating myoblasts (Mb) with 48 hr suspension arrested myoblasts (G0 Mb). Green boxes represent genes down-regulated in G0 and red boxes represent genes upregulated in G0 (white boxes surround genes that participate in the cell cycle but were not spotted on the array). Expectedly the entire cell cycle network is suppressed coincident with the induction of genes such as the tumor suppressor p53 and the TGFb target transcription factor Smad3 (see Figure S2 for TGFb pathway induced in G0). Interestingly, the Orc4l DNA binding subunit of the Origin Replication Complex is induced, perhaps indicating a mechanism that marks origins in reversibly quiescent cells. (TIF) [file pone.0065097.s001.tif]

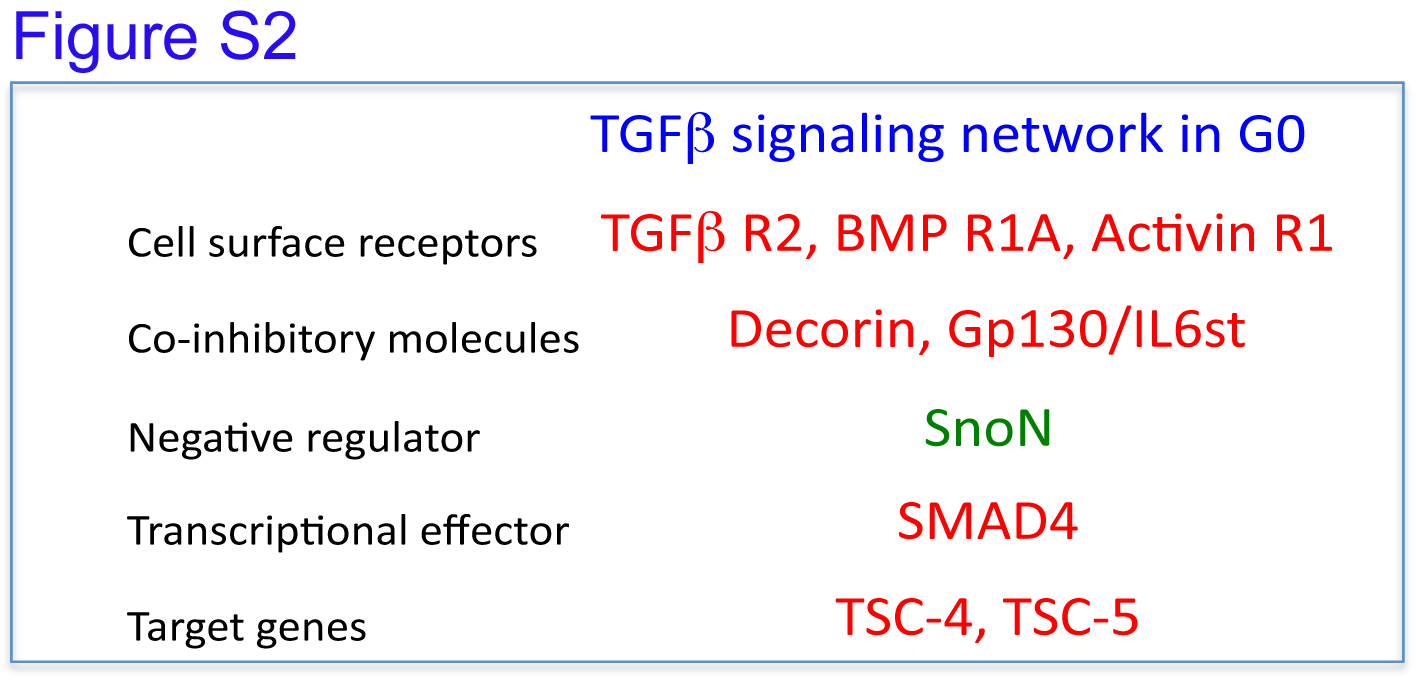

Supplement: Figure S2 — The TGFb pathway is induced in G0 myoblasts. The TGF-b signaling network is up-regulated in quiescent myoblasts. Genes positively induced include participants at all levels of the pathway including cell surface receptors, co-receptors, transcriptional effectors and target genes. Negative regulator SnoN is repressed in quiescent myoblasts, indicating an overall induction of TGFb signaling, a known participant in quiescence and repression of myogenesis. (TIF) [file pone.0065097.s002.tif]

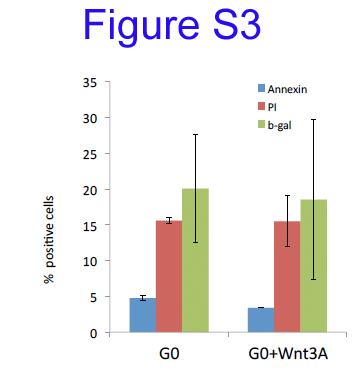

Supplement: Figure S3 — Wnt 3A treatment of quiescent myoblasts does not induce either apoptosis or senescence. The negative effects of Wnt on clonogenic self-renewal (Figure 8A) were not a result of induction of cell death or senescence pathways. Myoblasts were cultured in methocel suspension for 48 hours in the absensce (G0) or in the presence (G0+ Wnt3A). Both cultures were harvested and either stained for cell surface Annexin 5 or propidium iodine (PI) and analysed by flow cytometry. Senescence associated b-galactosidase activity was detected by cytological staining using a chromogenic substrate. Wnt 3A treatment of quiescent myoblasts does not increase rates of either apoptosis or senescence. (TIF) [file pone.0065097.s003.tif]

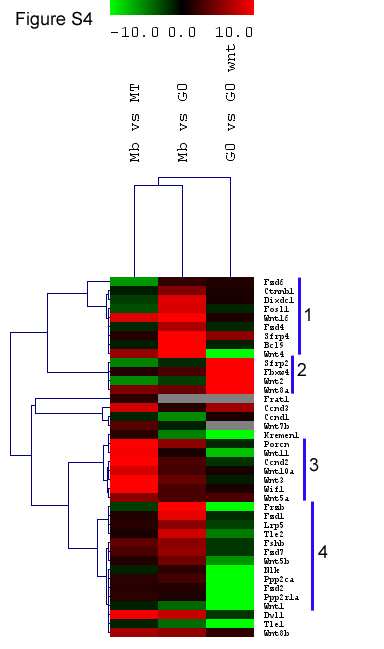

Supplement: Figure S4 — Wnt3A treatment of quiescent myoblasts drastically alters expression of the Wnt module. Hierarchical clustering of Wnt super-array data reveals that Wnt3A treatment of quiescent myoblasts drastically alters expression of the Wnt module. Four clusters of genes were readily discerned: (1) Genes strongly induced specifically in G0 but not in MT, and repressed in response to Wnt 3A. (2) Genes suppressed in G0 and strongly induced by Wnt3A. (3) Genes strongly induced in MT, mildly induced in G0 (common to two states of arrest), but suppressed by Wnt3A. (4) Genes mildly induced in G0 and strongly suppressed by Wnt3A. The alteration of nearly all genes in the Wnt module by enhanced Wnt signaling suggests the operation of the Wnt feedback control mechanism. (TIF) [file pone.0065097.s004.tif]

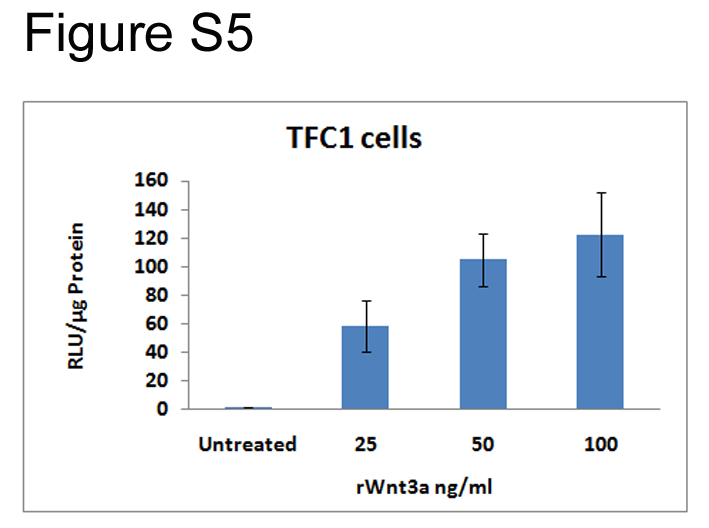

Supplement: Figure S5 — Dose response of Wnt3a treatment on TOPflash activity. Stably transfected Wnt reporter myoblasts (TFC1) were treated with different doses of Wnt3A (10, 50, 100 ng/ml) and TOPflash luciferase activity measured after 48 hours. 50 ng/ml elicited nearly as strong a response as 100 and was chosen for further experiments. (TIF) [file pone.0065097.s005.tif]
