# Supplementary material for: Distinct Transcriptional Networks in Quiescent Myoblasts: A Role for Wnt Signaling in Reversible vs. Irreversible Arrest
Source: PLoS One. 2013 Jun 3;8(6):e65097. doi: 10.1371/journal.pone.0065097 (PMC3670900; doi:10.1371/journal.pone.0065097)
Supplement: Table S1 — Selected Genes induced in G0 myoblasts. Based on Gene ontology searches, genes induced in quiescent myoblasts were classified into different functional classes. A partial list of the ∼1100 quiescence-induced genes is presented. (DOC) [file pone.0065097.s006.doc]

| **Unigene ID** | **Name** | **Symbol** | **Fold Change** |
| --- | --- | --- | --- |
| **A. DNA Repair** | | | |
| Mm.246952 | X-ray repair complementing defective repair in Chinese hamster cells 5 | Xrcc5 | 2.33 |
| Mm.287837 | Excision repair cross-complementing rodent repair deficiency, complementation group 4 | Ercc4 | 3.63 |
| Mm.322843 | Interferon-stimulated protein | Isg20 | 4.31 |
| Mm.335937 | Nonhomologous end-joining factor 1 | Nhej1 | 3.72 |
| Mm.100622 | Three prime repair exonuclease 1 | Trex1 | 3.01 |
| Mm.291179 | 5'-3' exoribonuclease 1 | Xrn1 | 2.02 |
| Mm.116968 | AlkB, alkylation repair homolog 8 | Alkbh8 | 2.3 |
| Mm.89772 | DNA methyltransferase 3B | Dnmt3b | 3.28 |
| Mm.6536 | Fanconi anemia, complementation group A | Fanca | 2.15 |
| Mm.195635 | Single-stranded DNA binding protein 3 | Ssbp3 | 3.2 |
| Mm.305816 | Prostaglandin E synthase 3 (cytosolic) | Ptges3 | 2.07 |
| Mm.11333 | Regulator of telomere elongation helicase 1 | Rtel1 | 1.72 |
| Mm.6402 | Telomeric repeat binding factor 2 | Terf2 | 3.94 |
| Mm.434328 | Tax1 (human T-cell leukemia virus type I) binding protein 1 | Tax1bp1 | 1.67 |
| Mm.249310 | Tankyrase, TRF1-interacting ankyrin-related ADP-ribose polymerase 2 | Tnks2 | 1.64 |
| Mm.254820 | Single-strand selective monofunctional uracil DNA glycosylase | Smug1 | 1.66 |
| Mm.422265 | Zinc finger, RAN-binding domain containing 3 | Zranb3 | 5.93 |
| **B. Redox Pathway** | | | |
| Mm.247542 | Peroxiredoxin 4 | Prdx4 | 2.79 |
| Mm.279782 | Peroxiredoxin 5 | Prdx5 | 1.69 |
| Mm.282711 | Peroxisomal biogenesis factor 3 | Pex3 | 2.39 |
| Mm.247764 | Peroxisome biogenesis factor 19 | Pex19 | 2.55 |
| Mm.410189 | Thioredoxin interacting protein | Txnip | 9.19 |
| Mm.268041 | Thioredoxin domain containing 10 | Txndc10 | 3.23 |
| Mm.37199 | Glutathione S-transferase, mu 1 | Gstm1 | 2.89 |
| Mm.2662 | Glutathione S-transferase, alpha 4 | Gsta4 | 5.24 |
| Mm.276389 | Heme oxygenase (decycling) 1 | Hmox1 | 2.41 |
| Mm.11223 | Xanthine dehydrogenase | Xdh | 1.61 |
| Mm.46346 | NHL repeat containing 2 | Nhlrc2 | 2.79 |
| Mm.179069 | A kinase (PRKA) anchor protein 7 | Akap7 | 2.64 |
| Mm.192991 | Metallothionein 1 | Mt1 | 1.72 |
| Mm.379011 | Sterol carrier protein 2, liver | Scp2 | 2.39 |
| Mm.370024 | RIKEN cDNA 4930402E16 gene | 4930402E16Rik | 5.98 |
| **C. Autophagy** | | | |
| Mm.271898 | Unc-51 like kinase 1 (C. elegans) | Ulk1 | 1.9 |
| Mm.9852 | Autophagy-related 12 (yeast) | Atg12 | 1.7 |
| Mm.196239 | Microtubule-associated protein 1 light chain 3 alpha | Map1lc3a | 4.5 |
| Mm.28357 | Microtubule-associated protein 1 light chain 3 beta | Map1lc3b | 2.7 |
| Mm.210745 | Glutamate-ammonia ligase (glutamine synthetase) | Glul | 7.1 |
|  |  |  |  |
| **D. Ubiquitin pathway** | | | |
| Mm.331 | Ubiquitin C | Ubc | 2.23 |
| Mm.332967 | Ubiquitin-conjugating enzyme E2H | Ube2h | 2.29 |
| Mm.305925 | Ariadne ubiquitin-conjugating enzyme E2 binding protein homolog 1 | Arih1 | 2.19 |
| Mm.297706 | Zinc finger CCCH type containing 5 | Zc3h5 | 2.99 |
| Mm.191892 | Hect domain and RLD 3 | Herc3 | 2.38 |
| Mm.41864 | Ubiquitin-associated protein 2 | Ubap2 | 2.47 |
| Mm.426531 | Transformed mouse 3T3 cell double minute 4 | Mdm4 | 2.4 |
| Mm.102496 | Nedd4 family interacting protein 1 | Ndfip1 | 1.63 |
| Mm.242646 | Ubiquitin specific peptidase 9, X chromosome | Usp9x | 1.63 |
| Mm.87611 | Cullin 1 | Cul1 | 1.82 |
| Mm.319123 | F-box and leucine-rich repeat protein 13 | Fbxl13 | 2.32 |
| Mm.390165 | F-box and leucine-rich repeat protein 12 | Fbxl12 | 2.21 |
| Mm.6370 | Protein inhibitor of activated STAT 2 | Pias2 | 1.74 |
| Mm.262859 | Ring finger protein 38 | Rnf38 | 1.93 |
| Mm.427762 | Ring finger protein 12 | Rnf12 | 1.7 |
| Mm.386792 | Zinc finger protein 364 | Zfp364 | 2.04 |
| Mm.245210 | Leucine-rich repeats and immunoglobulin-like domains 1 | Lrig1 | 3.33 |
| Mm.275426 | E3 ubiquitin protein ligase, HECT domain containing, 1 | Edd1 | 1.73 |
| Mm.390895 | YEATS domain containing 2 | Yeats2 | 2.33 |
| **E. Anti-apoptosis** | | | |
| Mm.297482 | Tumor protein, translationally-controlled 1 | Tpt1 | 1.93 |
| Mm.290908 | Baculoviral IAP repeat-containing 6 | Birc6 | 2.33 |
| Mm.26840 | Neural regeneration protein | Nrp | 2.54 |
| Mm.27432 | DnaJ (Hsp40) homolog, subfamily B, member 9 | Dnajb9 | 2.14 |
| Mm.260521 | Phosphatidylinositol 3-kinase, catalytic, alpha polypeptide | Pik3ca | 3.07 |
| Mm.294783 | BCL2-associated transcription factor 1 | Bclaf1 | 2.26 |
| Mm.118400 | BCL2-associated athanogene 4 | Bag4 | 1.92 |
| Mm.246990 | Reticulon 3 | Rtn3 | 4.76 |
| **F. Energy metabolism, glycolytic enzymes, solute transporters** | | | |
| Mm.379011 | Sterol carrier protein 2, liver | Scp2 | 2.11 |
| Mm.1514 | Lipoprotein lipase | Lpl | 7.71 |
| Mm.207004 | Protein kinase, AMP-activated, alpha 1 catalytic subunit | Prkaa1 | 2 |
| Mm.381 | Adipose differentiation related protein | Adfp | 3.07 |
| Mm.301527 | Pyruvate dehydrogenase (lipoamide) beta | Pdhb | 4.22 |
| Mm.34411 | Pyruvate dehydrogenase kinase, isoenzyme 1 | Pdk1 | 3.86 |
| Mm.196605 | Hexokinase 1 | Hk1 | 2.28 |
| Mm.9925 | Isocitrate dehydrogenase 1 (NADP+), soluble | Idh1 | 3.17 |
| Mm.119320 | Carbonic anhydrase 8 | Car8 | 2.53 |
| Mm.141230 | 1-acylglycerol-3-phosphate O-acyltransferase 3 | Agpat3 | 3.04 |
| Mm.620 | Arylsulfatase A | Arsa | 2.88 |
| Mm.18905 | Mannosidase, alpha, class 1C, member 1 | Man1c1 | 2.76 |
| Mm.210745 | Glutamate-ammonia ligase (glutamine synthetase) | Glul | 7.11 |
| Mm.268841 | Threonine synthase-like 1 (bacterial) | Thnsl1 | 2.44 |
| Mm.27844 | ATP synthase, H+ transporting, mitochondrial F0 complex, subunit s | Atp5s | 2.43 |
| Mm.41651 | ATP synthase mitochondrial F1 complex assembly factor 2 | Atpaf2 | 1.69 |
| Mm.21002 | Solute carrier family 2 (facilitated glucose transporter), member 1 | Slc2a1 | 8.91 |
| Mm.293635 | Solute carrier family 25, member 37 | Slc25a37 | 3.01 |
| Mm.432747 | Solute carrier family 2 (facilitated glucose transporter), member 3 | Slc2a3 | 3.02 |
| Mm.261614 | Solute carrier family 12, member 6 | Slc12a6 | 2.8 |
| Mm.103581 | Solute carrier family 23 (nucleobase transporters), member 2 | Slc23a2 | 2.54 |
| Mm.17875 | Solute carrier family 25, member 32(Mitochondrial folate transporter) | Slc25a32 | 2.49 |
| Mm.27801 | Solute carrier family 30 (zinc transporter), member 4 | Slc30a4 | 2.29 |
| Mm.281804 | Solute carrier family 15 (H+/peptide transporter), member 2 | Slc15a2 | 2.09 |
| Mm.217764 | Phosphoglucomutase 2 | Pgm2 | 1.76 |
| **G. Chromatin re-modeling** | | | |
| Mm.260479 | Jumonji domain containing 1A | Jmjd1a | 4.07 |
| Mm.45047 | Jumonji domain containing 2B | Jmjd2b | 4.75 |
| Mm.25059 | Jumonji, AT rich interactive domain 2 | Jarid2 | 3.14 |
| Mm.333357 | Cat eye syndrome chromosome region, candidate 2 homolog | Cecr2 | 4.2 |
| Mm.157190 | High mobility group AT-hook 2 | Hmga2 | 2.55 |
| Mm.402739 | High mobility group 20A | Hmg20a | 1.64 |
| Mm.390461 | High mobility group box transcription factor 1 | Hbp1 | 3.83 |
| Mm.41447 | Chromodomain helicase DNA binding protein 1-like | Chd1l | 1.62 |
| Mm.27913 | Polybromo 1 | Pb1 | 3.04 |
| Mm.252213 | Bromodomain adjacent to zinc finger domain, 2A | Baz2a | 2.19 |
| Mm.5400 | Bromodomain containing 7 | Brd7 | 1.6 |
| Mm.192111 | SET domain containing (lysine methyltransferase) 7 | Setd7 | 2.41 |
| Mm.288949 | SET domain containing 2 | Setd2 | 2.42 |
| Mm.242791 | Suppressor of hairy wing homolog 4 | Suhw4 | 2.81 |
| Mm.205400 | MYST histone acetyltransferase 2 | Myst2 | 2.19 |
| Mm.275044 | Lin-9 homolog | Lin9 | 2.01 |
| Mm.426209 | Mortality factor 4 like 1 | Morf4l1 | 1.67 |
| Mm.71682 | Calcium binding and coiled coil domain 1 | Calcoco1 | 3.68 |
| Mm.295670 | Chromatin modifying protein 2A | Chmp2a | 2.25 |
| Mm.262480 | Chromatin modifying protein 4B | Chmp4b | 1.86 |
| Mm.301039 | Nuclear receptor coactivator 1 | Ncoa1 | 2.52 |
| Mm.271814 | Nuclear receptor co-repressor 1 | Ncor1 | 1.91 |
| Mm.18742 | Nuclear protein 1 | Nupr1 | 1.74 |
| Mm.132238 | CREB binding protein | Crebbp | 1.63 |
| Mm.15694 | Histone cell cycle regulation defective homolog A | Hira | 1.82 |
| Mm.24350 | H1 histone family, member 0 | H1f0 | 2.28 |
| Mm.20521 | Histone deacetylase 3 | Hdac3 | 2.93 |
| Mm.132868 | Retinoblastoma binding protein 5 | Rbbp5 | 1.86 |
| Mm.24761 | Breast cancer metastasis-suppressor 1-like | Brms1l | 2.03 |
| **H. Transcription Factors** | | | |
| Mm.278444 | Transducin-like enhancer of split 1, homolog of Drosophila E(spl) | Tle1 | 2.51 |
| Mm.425101 | EP300 interacting inhibitor of differentiation 1 | Eid1 | 2.51 |
| Mm.28184 | Myocyte enhancer factor 2D | Mef2d | 1.66 |
| Mm.1025 | Nuclear factor, erythroid derived 2, like 2 | Nfe2l2 | 1.88 |
| Mm.276133 | LUC7-like 2 | Luc7l2 | 2.05 |
| Mm.390057 | Nuclear factor of activated T-cells 5 | Nfat5 | 3.15 |
| Mm.265917 | Thyroid hormone receptor alpha | Thra | 3.9 |
| Mm.247566 | Myeloid ecotropic viral integration site-related gene 1 | Mrg1 | 4.11 |
| Mm.356578 | Myeloid ecotropic viral integration site 1 | Meis1 | 2.47 |
| Mm.4325 | Kruppel-like factor 4 (gut) | Klf4 | 3.54 |
| Mm.291595 | Kruppel-like factor 9 | Klf9 | 4.43 |
| Mm.38193 | Zinc finger protein 292 | Zfp292 | 3.59 |
| Mm.26594 | Zinc finger protein 508 | Zfp508 | 3.03 |
| Mm.276296 | Zinc finger protein 422, related sequence 1 | Zfp422-rs1 | 3.71 |
| Mm.290924 | Zinc finger protein 91 | Zfp91 | 2.1 |
| Mm.39496 | Zinc finger protein, multitype 2 | Zfpm2 | 1.99 |
| Mm.251083 | Zinc finger protein 84 | Zfp84 | 2.1 |
| Mm.195877 | Zinc finger protein 106 | Zfp106 | 1.88 |
| Mm.87487 | Zinc finger protein 612 | Zfp612 | 1.93 |
| Mm.386792 | Zinc finger protein 364 | Zfp364 | 2.04 |
| Mm.27575 | Zinc finger protein 68 | Zfp68 | 2.01 |
| Mm.286232 | Zinc finger protein 532 | Zfp532 | 4.99 |
| Mm.235132 | Zinc finger protein 36, C3H type-like 1 | Zfp36l1 | 3.96 |
| Mm.422265 | Zinc finger, RAN-binding domain containing 3 | Zranb3 | 5.93 |
| Mm.29891 | Forkhead box O1 | Foxo1 | 2.98 |
| Mm.87142 | Forkhead box J2 | Foxj2 | 2.28 |
| Mm.968 | Nuclear receptor subfamily 1, group H, member 2 | Nr1h2 | 2.54 |
| Mm.277533 | Yippee-like 5 | Ypel5 | 2.51 |
| Mm.27114 | Yippee-like 3 | Ypel3 | 3.61 |
| Mm.247272 | Nuclear factor of kappa light polypeptide gene enhancer in B-cells inhibitor, zeta | Nfkbiz | 2.38 |
| Mm.170515 | Nuclear factor of kappa light chain gene enhancer in B-cells inhibitor, alpha | Nfkbia | 3.12 |
| Mm.294783 | BCL2-associated transcription factor 1 | Bclaf1 | 2.26 |
| Mm.2018 | Core binding factor beta | Cbfb | 1.78 |
| Mm.132634 | Ras responsive element binding protein 1 | Rreb1 | 1.61 |
| Mm.271814 | Nuclear receptor co-repressor 1 | Ncor1 | 1.91 |
| **I. Mediator Complex** | | |  |
| Mm.260576 | Cyclin-dependent kinase 8 | Cdk8 | 7.36 |
| Mm.278584 | Cyclin C | Ccnc | 2.77 |
| Mm.279993 | Mediator of RNA polymerase II transcription, subunit 6 homolog | Med6 | 2.51 |
| Mm.236211 | Thyroid hormone receptor associated protein 3 | Thrap3 | 1.97 |
| Mm.11333 | Regulator of telomere elongation helicase 1 | Rtel1 | 1.73 |
| Mm.74982 | Cell cycle related kinase | Ccrk | 3.61 |
| Mm.255858 | Ctr9, Paf1/RNA polymerase II complex component, homolog | Ctr9 | 3.34 |
| Mm.24159 | Cofactor required for Sp1 transcriptional activation, subunit 9 | Crsp9 | 2.89 |
| **J. Unusual Cyclins (RNA pol II-associated)** | | | |
| Mm.278584 | Cyclin C | Ccnc | 2.76 |
| Mm.23492 | Cyclin L2 | Ccnl2 | 5.39 |
| Mm.329864 | Cyclin M1 | Cnnm1 | 1.74 |
| Mm.250419 | Cyclin I | Ccni | 2.1 |
| Mm.260576 | Cyclin-dependent kinase 8 | Cdk8 | 7.34 |
| Mm.74982 | Cell cycle related kinase | Ccrk | 3.61 |
| **K. Translation factors and RNA-binding proteins** | | | |
| Mm.29644 | DEAD (Asp-Glu-Ala-Asp) box polypeptide 17 | Ddx17 | 1.83 |
| Mm.296370 | DEAD (Asp-Glu-Ala-Asp) box polypeptide 5 | Ddx5 | 1.61 |
| Mm.260627 | DEAH (Asp-Glu-Ala-His) box polypeptide 40 | Dhx40 | 3.27 |
| Mm.260084 | Eukaryotic translation initiation factor 4A2 | Eif4a2 | 2.16 |
| Mm.251255 | DEAD (Asp-Glu-Ala-Asp) box polypeptide 1 | Ddx1 | 1.64 |
| Mm.294769 | Insulin-like growth factor 2 mRNA binding protein 1 | Igf2bp1 | 1.66 |
| Mm.281018 | Insulin-like growth factor 2 mRNA binding protein 3 | Igf2bp3 | 3.03 |
| Mm.320469 | PAN3 polyA specific ribonuclease subunit homolog | Pan3 | 1.61 |
| Mm.139926 | RNA binding motif protein 6 | Rbm6 | 3.78 |
| Mm.275106 | RNA binding motif protein 22 | Rbm22 | 2.45 |
| Mm.392436 | RNA binding motif protein 39 | Rbm39 | 2.34 |
| Mm.223946 | Splicing factor, arginine/serine-rich 11 | Sfrs11 | 2.56 |
| Mm.233855 | Eukaryotic translation initiation factor 2B, subunit 5 epsilon | Eif2b5 | 2.83 |
| Mm.260084 | Eukaryotic translation initiation factor 4A2 | Eif4a2 | 2.16 |
| Mm.182962 | Eukaryotic translation initiation factor 3, subunit 5 (epsilon) | Eif3s5 | 2.07 |
| Mm.3451 | Fragile X mental retardation syndrome 1 homolog | Fmr1 | 2.14 |
| Mm.259021 | Fragile X mental retardation gene 1, autosomal homolog | Fxr1h | 2.04 |
| Mm.29989 | SYF2 homolog, RNA splicing factor | Syf2 | 1.78 |
| Mm.20129 | Paraspeckle protein 1 | Pspc1 | 2.43 |
| Mm.61064 | Poly(A) polymerase gamma | Papolg | 2.07 |
| Mm.274466 | Poly (ADP-ribose) polymerase family, member 8 | Parp8 | 2.91 |
| Mm.398543 | CUG triplet repeat, RNA binding protein 2 | Cugbp2 | 4.22 |
| **L. Selected Cell Surface Receptors** | | | |
| Mm.265716 | Fibroblast growth factor receptor 1 | Fgfr1 | 8.84 |
| Mm.16340 | Fibroblast growth factor receptor 2 | Fgfr2 | 2.02 |
| Mm.7995 | Fibroblast growth factor 13 | Fgf13 | 2.63 |
| Mm.275742 | Insulin-like growth factor I receptor | Igf1r | 3.66 |
| Mm.26553 | Insulin-like growth factor 2 receptor | Igf2r | 2.88 |
| Mm.195877 | Zinc finger protein 106 | Zfp106 | 1.88 |
| Mm.42033 | Growth factor receptor bound protein 2-associated protein 2 | Gab2 | 3.37 |
| Mm.221403 | Platelet derived growth factor receptor, alpha polypeptide | Pdgfra | 6.16 |
| Mm.4146 | Platelet derived growth factor receptor, beta polypeptide | Pdgfrb | 1.66 |
| Mm.21013 | Chemokine (C-X-C motif) ligand 1 | Cxcl1 | 26.15 |
| Mm.38241 | Interleukin-1 receptor-associated kinase 1 | Irak1 | 7.93 |
| Mm.24208 | Interleukin 13 receptor, alpha 1 | Il13ra1 | 3.61 |
| Mm.425857 | Interleukin 3 receptor, alpha chain | Il3ra | 3.34 |
| Mm.38241 | Interleukin-1 receptor-associated kinase 1 | Irak1 | 3.1 |
| Mm.380801 | Interleukin 31 receptor A | Il31ra | 2.07 |
| Mm.269363 | Interleukin 17 receptor B | Il17rb | 1.62 |
| Mm.237825 | Bone morphogenetic protein receptor, type 1A | Bmpr1a | 2.93 |
| Mm.172346 | Transforming growth factor, beta receptor II | Tgfbr2 | 4.62 |
| Mm.153272 | TSC22 domain family, member 1 | Tsc22d1 | 2.24 |
| Mm.100399 | MAD homolog 4 | Smad4 | 2.1 |
| Mm.29798 | CD34 antigen | Cd34 | 3.79 |
| Mm.314338 | Activin receptor IIA | Acvr2a | 2.87 |
| Mm.15125 | Neuroplastin | Nptn | 2.11 |
| Mm.1682 | Signal-regulatory protein alpha | Sirpa | 2.26 |
| Mm.311110 | Integrin alpha 2b | Itga2b | 2.16 |
| Mm.6424 | Integrin beta 5 | Itgb5 | 2.01 |
| Mm.334685 | Integrin alpha FG-GAP repeat containing 1 | Itfg1 | 2.48 |
| Mm.425327 | Glutamate receptor, ionotropic, delta 2 | Grid2 | 4.29 |
| Mm.41665 | Glutamate receptor, ionotropic, N-methyl D-asparate-associated protein 1 (glutamate binding) | Grina | 4.41 |
| Mm.288282 | Activated leukocyte cell adhesion molecule | Alcam | 2.45 |
| Mm.269815 | CD164 antigen | Cd164 | 2.46 |
| Mm.806 | CD 81 antigen | Cd81 | 1.65 |
| Mm.4261 | CD82 antigen | Cd82 | 1.67 |
| Mm.271854 | Low density lipoprotein receptor-related protein 1 | Lrp1 | 2.65 |
| Mm.2976 | Podoplanin | Pdpn | 2.1 |
| **Embryonic signaling pathways** | | | |
| **M. Hedgehog pathway** | | | |
| Mm.3484 | Niemann Pick type C1 | Npc1 | 2.12 |
| Mm.22701 | Growth arrest specific 1 | Gas1 | 4.72 |
| Mm.423137 | Arrestin domain containing 3 | Arrdc3 | 4.09 |
| **N. BMP Pathway** | | | |
| Mm.10153 | Twisted gastrulation homolog 1 | Twsg1 | 2.93 |
| Mm.27757 | Bone morphogenetic protein 1 | Bmp1 | 3.1 |
| Mm.118034 | Bone morphogenetic protein 5 | Bmp5 | 2.06 |
| Mm.237825 | Bone morphogenetic protein receptor, type 1A | Bmpr1a | 2.92 |
| **O. Notch Pathway** | | | |
| Mm.293761 | Protein O-fucosyltransferase 1 | Pofut1 | 4.44 |
| Mm.27681 | A disintegrin and metallopeptidase domain 17 | Adam17 | 3.49 |
| Mm.170515 | Nuclear factor of kappa light chain gene enhancer in B-cells inhibitor, alpha | Nfkbia | 3.1 |
| Mm.209292 | Recombining binding protein suppressor of hairless | Rbpsuh | 2.69 |
| Mm.262102 | Strawberry notch homolog 2 | Sbno2 | 2.49 |
| Mm.4364 | Interleukin 6 signal transducer | Il6st | 2.4 |
| Mm.260576 | Cyclin-dependent kinase 8 | Cdk8 | 7.34 |
| Mm.149235 | Manic fringe homolog | Mfng | 2.51 |
| Mm.21500 | Mindbomb homolog 1 | Mib1 | 3.56 |
