# Supplementary material for: Distinct Transcriptional Networks in Quiescent Myoblasts: A Role for Wnt Signaling in Reversible vs. Irreversible Arrest
Source: PLoS One. 2013 Jun 3;8(6):e65097. doi: 10.1371/journal.pone.0065097 (PMC3670900; doi:10.1371/journal.pone.0065097)
Supplement: Table S2 — Genes commonly enriched in Quiescent C2C12 myoblasts (this study) and freshly isolated muscle satellite cells (Fukuda et al, 2007). Based on comparison of the data generated in this study (1.6 fold up-regulated in G0) with the data generated from freshly isolated mouse SC (Fukada et al, 2007; 5-fold up-regulated in G0), a list of commonly G0-induced genes is presented. Note the common induction of SC markers CD34 and Sca1, Wnt regulator Rgs2, signaling components Stat3 and Decorin, Stem cell marker Klf4, all indicating a shared network in quiescent cells in vitro and in vivo. (DOC) [file pone.0065097.s007.doc]

| **Unigene ID** | **Name** | **Symbol** |
| --- | --- | --- |
| Mm.29798 | CD34 antigen | Cd34 |
| Mm.28262 | Regulator of G-protein signaling 2 | Rgs2 |
| Mm.139418 | Sestrin 1 | Sesn1 |
| Mm.275071 | Jun oncogene | Jun |
| Mm.23492 | Cyclin L2 | Ccnl2 |
| Mm.330731 | Transglutaminase 2, C polypeptide | Tgm2 |
| Mm.9075 | Epoxide hydrolase 1, microsomal | Ephx1 |
| Mm.236553 | Cathepsin B | Ctsb |
| Mm.401675 | Tripeptidyl peptidase II | Tpp2 |
| Mm.261818 | Ring finger protein 167 | Rnf167 |
| Mm.426080 | Adducin 3 (gamma) | Add3 |
| Mm.273584 | RIKEN cDNA 4632428N05 gene | 4632428N05Rik |
| Mm.425949 | Lymphocyte antigen 6 complex, locus A | Ly6a, Sca1 |
| Mm.3303 | Fc receptor, IgG, alpha chain transporter | Fcgrt |
| Mm.265716 | Fibroblast growth factor receptor 1 | Fgfr1 |
| Mm.21013 | Chemokine (C-X-C motif) ligand 1 | Cxcl1 |
| Mm.56769 | Decorin | Dcn |
| Mm.392203 | Selenoprotein P, plasma, 1 | Sepp1 |
| Mm.4871 | Tissue inhibitor of metalloproteinase 3 | Timp3 |
| Mm.2509 | Procollagen, type VI, alpha 1 | Col6a1 |
| Mm.4258 | Osteoglycin | Ogn |
| Mm.29564 | Matrix metallopeptidase 2 | Mmp2 |
| Mm.1421 | A disintegrin-like and metallopeptidase(reprolysin type) with thrombospondin type 1 motif, 1 | Adamts1 |
| Mm.14455 | Transforming growth factor, beta induced | Tgfbi |
| Mm.249555 | Procollagen, type III, alpha 1 | Col3a1 |
| Mm.1249 | Laminin, gamma 1 | Lamc1 |
| Mm.277792 | Procollagen, type I, alpha 2 | Col1a2 |
| Mm.8655 | Complement component factor h | Cfh |
| Mm.154457 | TAP binding protein | Tapbp |
| Mm.17629 | Caspase recruitment domain family, member 10 | Card10 |
| Mm.30837 | N-myc downstream regulated gene 1 | Ndrg1 |
| Mm.101946 | Nuclear receptor binding protein 2 | Nrbp2 |
| Mm.192991 | Metallothionein 1 | Mt1 |
| Mm.298256 | Metastasis associated lung adenocarcinoma transcript 1 (non-coding RNA) | Malat1 |
| Mm.26722 | N-myc downstream regulated gene 2 | Ndrg2 |
| Mm.28357 | Microtubule-associated protein 1 light chain 3 beta | Map1lc3b |
| Mm.29389 | Tensin like C1 domain-containing phosphatase | Tenc1 |
| Mm.23963 | Phospholipase C, delta 1 | Plcd1 |
| Mm.27769 | Proline-rich nuclear receptor coactivator 1 | Pnrc1 |
| Mm.170515 | Nuclear factor of kappa light chain gene enhancer in B-cells inhibitor, alpha | Nfkbia |
| Mm.246398 | TCDD-inducible poly(ADP-ribose) polymerase | Tiparp |
| Mm.1639 | Myeloid cell leukemia sequence 1 | Mcl1 |
| Mm.348326 | Phosphatidic acid phosphatase type 2B | Ppap2b |
| Mm.291595 | Kruppel-like factor 9 Nuclear factor of kappa light polypeptide | Klf9 |
| Mm.247272 | gene enhancer in B-cells inhibitor, zeta | Nfkbiz |
| Mm.235132 | Zinc finger protein 36, C3H type-like 1 | Zfp36l1 |
| Mm.4325 | Kruppel-like factor 4 (gut) | Klf4 |
| Mm.181959 | Early growth response 1 | Egr1 |
| Mm.265917 | Thyroid hormone receptor alpha | Thra |
| Mm.31274 | Nuclear factor I/A | Nfia |
| Mm.5068 | Sorbin and SH3 domain containing 3 | Sorbs3 |
| Mm.249934 | Signal transducer and activator of transcription 3 | Stat3 |
| Mm.29891 | Forkhead box O1 | Foxo1 |
| Mm.25059 | Jumonji, AT rich interactive domain 2 | Jarid2 |
| Mm.410189 | Thioredoxin interacting protein | Txnip |
| Mm.281804 | Solute carrier family 15 (H+/peptide transporter),member 2 | Slc15a2 |
| Mm.21109 | Gelsolin | Gsn |
| Mm.300095 | UDP glucuronosyltransferase 1 family, polypeptide A7C | Ugt1a7c |
| Mm.395108 | Solute carrier family 2 (facilitated glucose transporter), 3 | Slc2a3 |
| Mm.14063 | Dehydrogenase/reductase (SDR family) member 3 | Dhrs3 |
| Mm.210745 | Glutamate-ammonia ligase (glutamine synthetase) | Glul |
| Mm.287807 | GM2 ganglioside activator protein | Gm2a |
| Mm.436932 | RNA binding motif protein 39 | Rbm39 |
| Mm.276133 | LUC7-like 2 (S. cerevisiae) | Luc7l2 |
| Mm.245210 | Leucine-rich repeats and immunoglobulin­ like domains 1 | Lrig1 |
| Mm.27114 | Yippee-like 3 (Drosophila) | Ypel3 |
| Mm.181074 | Coiled-coil domain containing 80 | Ccdc80 |
| Mm.29997 | Reticulocalbin 3, EF-hand calcium binding domain | Rcn3 |
| Mm.6424 | Integrin beta 5 | Itgb5 |
| Mm.22701 | Growth arrest specific 1 | Gas1 |
| Mm.4146 | Platelet derived growth factor receptor, beta polypeptide | Pdgfrb |
| Mm.386750 | Rho GTPase activating protein 6 | Arhgap6 |
