# Supplementary material for: Distinct Transcriptional Networks in Quiescent Myoblasts: A Role for Wnt Signaling in Reversible vs. Irreversible Arrest
Source: PLoS One. 2013 Jun 3;8(6):e65097. doi: 10.1371/journal.pone.0065097 (PMC3670900; doi:10.1371/journal.pone.0065097)
Supplement: Table S3 — Wnt pathway gene expression in proliferating myoblasts, myotubes, G0 myoblasts and G0 myoblasts treated with Wnt 3A [50 ng/ml]. Alteration of the Wnt module by treatment of G0 myoblasts with the ligand Wnt3a suggesting feedback control. (DOC) [file pone.0065097.s008.doc]

**Subramaniam et al. Table S3 Comparison of Wnt pathway genes- transcript profiling by SuperArray (Q-RT-PCR)**

Values represent fold change in mRNA levels and reflect the induction or repression in the second sample in each sample pair (Mb vs MT; Mb vs G0; G0 vs G0+Wnt). Values shown in red are up-regulated in the second sample, those in blue are down-regulated. For example, Frzb is minimally down-regulated in MT compared to Mb, strongly up-regulated in G0 compared to Mb and strongly down-regulated in G0+Wnt compared to G0, suggesting that Frzb is strongly induced in a quiescence-dependent manner and treatment of quiescent cells with Wnt leads to its suppression and by inference, subversion of a quiescence program.

| **Symbol** | **Mb vs MT** | **Mb vs G0** | **G0 vs G0 + Wnt** |
| --- | --- | --- | --- |
| Bcl9 | -1.1482 | 16.0834 | -1.2874 |
| Ccnd1 | -1.3858 | -5.479 | 1.0542 |
| Ccnd2 | 12.1883 | 2.9804 | -2.0007 |
| Ccnd3 | 8.1417 | 1.5762 | 6.2581 |
| Ctnnb1 | -1.0757 | 5.1005 | 1.2031 |
| Dixdc1 | -2.4874 | 8.7827 | 1.0315 |
| Dvl1 | 9.5479 | 7.8204 | -2.172 |
| Fbxw4 | 1.5535 | 2.9266 | 158.064 |
| Fosl1 | -3.0872 | 8.4571 | -1.5215 |
| Frat1 | 1.7365 |  |  |
| Frzb | -2.407 | 826.6749 | -324.1178 |
| Fshb | 3.8386 | 5.4197 | -2.1761 |
| Fzd1 | 1.3466 | 9.2836 | -1.662 |
| Fzd2 | 1.5589 | 1.4376 | -26.5728 |
| Fzd4 | -1.6084 | 6.8001 | -1.5489 |
| Fzd6 | -5.9439 | 1.958 | 1.3694 |
| Fzd7 | 2.9927 | 5.8026 | -2.1329 |
| Kremen1 | 1.6751 | -5.0499 | -12.9884 |
| Lrp5 | 1.5721 | 5.4154 | -2.5701 |
| Nlk | -1.3273 | 1.9137 | -41.7938 |
| Porcn | 11.2637 | 5.5585 | -1.5875 |
| Ppp2ca | 1.6975 | 2.7549 | -14.6171 |
| Ppp2r1a | 1.8169 | 1.2073 | -17.3141 |
| Sfrp2 | -5.207 | -1.6806 | 10.3054 |
| Sfrp4 | 1.385 | 209.3895 | 5.6185 |
| Tle1 | -1.3607 | -4.2233 | -22.5828 |
| Tle2 | 1.093 | 8.118 | -4.9269 |
| Wif1 | 92.4088 | 2.8235 | 1.0769 |
| Wnt1 | -1.315 | -4.0883 | -121.4344 |
| Wnt10a | 8.4139 | 2.7248 | 1.0003 |
| Wnt11 | 9.6414 | 1.1229 | -7.6299 |
| Wnt16 | 8.6824 | 87.9685 | 1.2048 |
| Wnt2 | -5.4438 | -2.4918 | 1444.3279 |
| Wnt3 | 26.9436 | 4.1146 | -1.1585 |
| Wnt4 | 5.9931 | 645.325 | -9.883 |
| Wnt5a | 5.2999 | 2.9876 | 2.9697 |
| Wnt5b | 1.449 | 4.3681 | -5.7801 |
| Wnt7b | 3.4394 | -1.319 |  |
| Wnt8a | 4.9588 | 4.1146 | 42.7687 |
| Wnt8b | 6.4778 | 5.7104 | 1.895 |
