# Supplementary material for: Distinct Transcriptional Networks in Quiescent Myoblasts: A Role for Wnt Signaling in Reversible vs. Irreversible Arrest
Source: PLoS One. 2013 Jun 3;8(6):e65097. doi: 10.1371/journal.pone.0065097 (PMC3670900; doi:10.1371/journal.pone.0065097)
Supplement: Materials and Methods S1 — Information on antibodies, primers and si/shRNAs used in this study can be accessed in the Supporting Information Materials and Methods. (DOC) [file pone.0065097.s009.doc]

**Supporting Information**

***Subramaniam et al, Distinct Transcriptional networks in reversibly arrested myoblasts***

***Supporting Information Materials and Methods S1***

***S1a. Table of Primers used for Q-RT-PCR analysis***

***S1b. Table of Primers used for ChIP analysis***

***S1c. Table of Antibodies used for staining and ChIP analysis***

S1a. Primers used in this study

1. for transcript analysis

| Primer | Sequence |
| --- | --- |
| CyclinD1 F1 | AAGTGCGTGCAGAAGGAGATTGTG |
| CyclinD1 R1 | TCGGGCCGGATAGAGTTGTCAGT |
| Dkk3 F1 | GGGTCACTGCACCCAAAAGG |
| Dkk3 R1 | AGGCTCCAGTTCCCAGGTGA |
| GAPDH F1 | ATCAACCGGGAAGCCCATCAC |
| GAPDH R1 | CCTTTTGGCTCCACCCTTCA |
| Myf5F1 | CCCCACCTCCAACTGCTCTG |
| Myf5R1 | CCAAGCTGGACACGGAGCTT |
| MyoD F | AGCGTCTCGAAGGCCTCAT |
| MyoD R | AGCGCAGCTGAACAAGCTA |
| Myogenin F | TGGGCATGTAAGGTGTGTAAGA |
| Myogenin R | ACTTTAGGCAGCCGCTGGT |
| P21F1 | TCTCATGGTGTGGTGGAAAA |
| P21R1 | GCTGTGGCTGAAACACAAGA |
| Rgs2 F1 | TCCTGGGAAGCCCAAAACTG |
| Rgs2 R1 | CTGAATGCAGCCAGCCCATA |
| R-Spondin-F | ATCCACACGATGCAGGGACC |
| R-Spondin-R | CACGAAGGTCACTTTGGCGAT |

S1b. Primers for ChIP analysis :

| Name | Sequence |
| --- | --- |
| Β-Actin-800BP F | CAGCTTCTTTGCAGCTCCTT |
| B-Actin-800BP R | TAAAGTTGGCTGTGCCAGTG |
| B-Globin F | GACAAACATTATTCAGAGGGAGT |
| B-Globin R | AAGCAAATGTGAGGAGCAACTGAT |
| Myf5 57.5 kb F | TGTGGCTCTCTCTCCGTATG |
| Myf5 57.5 kb R | AATACAGACATGCAGGCTTCAC |
| MyoD DRR F1 | TCAGGACCAGGACCATGTCT |
| MyoD DRR R1 | CTGGACCTGTGGCCTCTTAC |
| Myog-80BP F | TATATTTATCTCTGGGTTCATG |
| Myog-80BP R | GCTCCCGCAGCCCCT |

S1c. ***Antibodies used in this study***

| **Antigen/Tag** | **Company** | **Cat#** | **Host/Target** | **IF** |
| --- | --- | --- | --- | --- |
| β-catenin | BD | 610154 | Mouse | 1:200 |
| BrdU | Roche | 555627 | Mouse | 1:400 |
| Desmin | Sigma | D 8281 | Rabbit | 1:50 |
| MHC | DHSB |  | Mouse | 1:10 |
| MyoD | Dako | M3512 | Mouse | 1:100 |
| Myogenin | Santa Cruz | SC-576 | Rabbit | 1:100 |
| P21 | BD | 556430 | Mouse | 1:200 |
| P27 | BD | 610241 | Mouse | 1:200 |
| HBP1 | Santa Cruz | SC25390 | Rabbit | 1:500 |
| IgG (IP) | Calbiochem |  | Rabbit | --------------- |
| Alexa 488 | Probes | A11034 | Goat α Rabbit | 1:500 |
| Alexa 594 | Probes | A11032 | Goat α Mouse | 1:500 |
| Alexa 594 | Probes | A11037 | Goat α Rabbit | 1:500 |
| Avidin 546 | Probes | S11225 |  | 1:200 |
| Avidin 488 | Probes | S11223 |  | 1:200 |
| Biotin-IgG | Vector | BA2000 | Goat α Mouse | 1:200 |
| Biotin-IgG | Vector | BA1000 | Goat α Rabbit | 1:200 |
